# m6A Modification Patterns With Distinct Immunity, Metabolism, and Stemness Characteristics in Soft Tissue Sarcoma

**DOI:** 10.3389/fimmu.2021.765723

**Published:** 2021-12-24

**Authors:** Zhen-Dong Huang, Lu-Lu Lin, Zi-Zhen Liu, Chao Hu, Hui-Yun Gu, Ren-Xiong Wei

**Affiliations:** ^1^ Department of Spine and Orthopedic Oncology, Zhongnan Hospital of Wuhan University, Wuhan, China; ^2^ Department of Stomatology, Southern Medical University, Guangzhou, China; ^3^ Department of Pathology and Pathophysiology, School of Basic Medicine, Wuhan University, Wuhan, China; ^4^ The Third Clinical School, Hubei University of Medicine, Shiyan, China

**Keywords:** m6A methylation, soft tissue sarcoma, cancer molecular subtypes, tumor microenvironment, immunotherapy

## Abstract

N6-methyladenosine (m6A) RNA methylation has been shown to have prognostic value in cancer. Nonetheless, its potential role regarding immunity, metabolism, and stemness in soft tissue sarcoma (STS) remains unknown. We comprehensively estimated the m6A modification patterns and corresponding immunity, metabolism, and stemness characteristics based on 568 STS samples and 21 m6A regulators. The m6Ascore was constructed to quantify m6A modification patterns in individuals using machine learning algorithms. Two distinct m6A modification patterns among the STS patients were identified, which exhibited differences in prognosis, immune cell infiltration, metabolic pathways, stemness, somatic mutation, and copy number variation. Thereafter, immunity-, metabolism-, and stemness phenotype-related genes associated with m6A modification were identified. Furthermore, patients with lower m6Ascores had increased antitumor immune responses, survival benefit under immunotherapy, tumor mutation burden, immunogenicity, and response to anti-PD-1/L1 immunotherapy. Immunotherapy sensitivity was validated using the IMvigor210 dataset. STS patients with lower m6Ascore might be more sensitive to docetaxel and gemcitabine. Finally, pan-cancer analysis illustrated the significant correlations of m6Ascore with clinical outcomes, immune cell infiltration, metabolism, and stemness. This study revealed that m6A modification plays an important role in immunity, metabolism, and stemness in STS. Evaluating the m6A modification pattern and development of m6Ascore may help to guide more effective immunotherapy and chemotherapy strategies.

## Introduction

Soft tissue sarcoma (STS) is a type of malignant tumor that originates from mesenchymal tissues. Compared to other cancers (such as tumors of the respiratory or digestive system), STS has a lower incidence rate and higher heterogeneity (1). Despite recent advances in diagnosis, molecular characterization and combination chemotherapy regimens, there are still great challenges in STS management, especially regarding improving the clinical outcomes, due to STS’s complexity and heterogeneity (2). In recent years, given the growing evidence that the immune system plays an important role in cancer progression and the encouraging results of immunotherapy in some types of cancers, such as non-small cell lung cancer (NSCLC) (3) and melanoma (4), it was thought to extend immunotherapy to sarcomas (5). Although immunotherapy is a promising cancer treatment, its response rate remains low (5). This is especially the case in STS, due to its extensive heterogeneity and unclear characterization of the tumor microenvironment (TME) in the molecular subtypes. Therefore, in-depth research on the role of these subtypes in predicting responses to immunotherapy in STS is needed.

N^6^-methyladenosine (m6A) has a key role in current tumor research (6). m6A methylation research may provide new insights to improve cancer treatment, and m6A methylation is also a significant prognostic biomarker (7, 8). m6A methylation is an important RNA modification and a common post-transcriptional modification of mRNA (9). The regulation of m6A methylation is mediated by methyltransferases (writers), demethylases (erasers), and m6A‐binding proteins (readers), which can contribute to the post‐transcriptional regulation of gene expression at the RNA level without altering base sequences (10). Some studies have revealed that m6A regulators are related to typical carcinogenic pathways. The m6A writer METTL3 promotes bladder cancer cell proliferation in an m6A-dependent manner by promoting the maturation of pre-miR221/222 (11). The m6A eraser ALKBH5 prevents pancreatic cancer progression by transcriptional activation of PER1 in an m6A-YTHDF2-dependent manner (12). As an oncogene, the m6A eraser FTO promotes IDH mutations through the FTO/MYC/CEBPA signaling pathway, which leads to tumorigenesis (13). Recent studies also revealed that immunotherapy is affected by m6A modification *via* changes in the TME and CD8+ T cell recruitment (14, 15). Research has highlighted that m6A modification plays an important role in cancer biology and tumor stemness (16). From this perspective, analysis of m6A modification could broaden the understanding of the mechanisms underlying STS occurrence and progression, while providing new insights into the clinical use of immunotherapy.

In this study, multiomics and clinical data of 568 STS samples were used to comprehensively identify distinct m6A modification patterns, and three important tumor characteristics (immunity, metabolism, and stemness) were assessed. In addition, the m6Ascore was developed using machine learning algorithms to quantify individual differences among different STS subtypes. m6Ascore was shown to predict responses to immunotherapy and chemotherapy. Finally, a pan-cancer analysis illustrated significant correlations of m6Ascore with prognosis, immune cell infiltration, metabolism, and stemness in other cancers, which indicated that it may help to guide the use of immunotherapy and chemotherapy in other cancers.

## Methods

The method details are described in the **Supplementary Methods**.

## Results

### Landscape of m6A Regulators in STS

**Figure 1:**
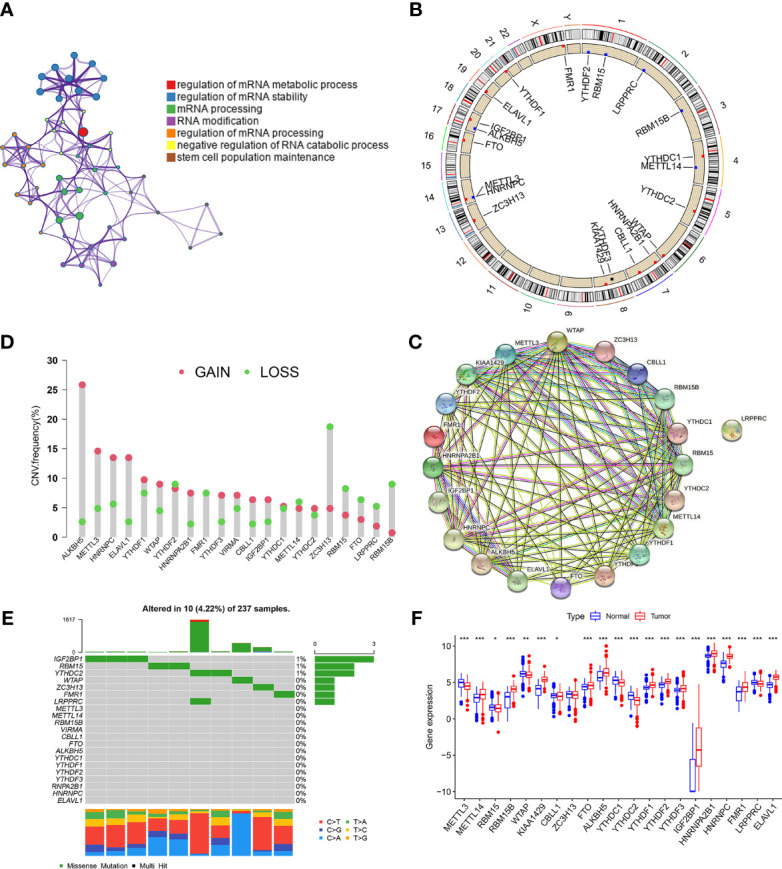
Landscape of m6A regulators in STS. **(A)** GO enrichment plot showing seven important terms related with m6A regulators. **(B)** The location of CNV alteration of m6A regulators on 23 chromosomes in TCGA-SARC cohorts. **(C)** The CNV variation frequency of m6A regulators in TCGA-SARC corhort. The height of the column represented the alteration frequency. Green dot and red dot represented the deletion frequency and the amplification frequency, respectively. **(D)** The protein-protein interactions between 21 m6A regulators. **(E)** The mutation frequencies of m6A regulators in TCGA-STS cohort. Each column represented an individual sample. The upper barplot showed tumor mutation load and the number on the right indicated the mutation frequency in each regulator. **(F)** The expression of 21 m6A regulators between normal tissues from Genotype-Tissue Expression samples and STS tissue from TCGA-SARC cohort. The statistical difference was compared through the Kruskal–Wallis test. *P < 0.05; **P < 0.01; ***P < 0.001.

The detailed workflow for m6A patterns and subsequent analyses are shown in **Figure S1A**. 21 m6A methylation regulators (“writers”: CBLL1, KIAA1429, METTL14, METTL3, RBM15, RBM15B, WTAP, ZC3H13; “readers”: ELAVL1, FMR1, HNRNPA2B1, HNRNPC, IGF2BP1, LRPPRC, YTHDC1, YTHDC2, YTHDF1, YTHDF2, YTHDF3; and “erasers”: ALKBH5 and FTO) were analyzed in STS. Gene Ontology (GO) enrichment analyses of these regulators were conducted, and the significantly enriched biological processes are summarized in **Figure 1A**. The locations across the chromosomes of the copy number variation (CNV) of the regulators are shown in **Figure 1B**. CNV was very common and mostly involved amplification, though FMR1, ZC3H13, RBM15, FTO, LRPPRC, and RBM15B had a high frequency of deletion (**Figure 1C**). The interaction patterns among the 21 m6A regulators were also analyzed using the Search Tool for the Retrieval of Interacting Genes/​Proteins (STRING) database (**Figure 1D**). Among the 237 STS samples in the The Cancer Genome Atlas Program (TCGA) cohort, 10 mutations were identified, mutation frequency of the 21 m6A regulators was 4.22%. IGF2BP1 had the highest mutation frequency, followed by RBM15 and YTHDC2 (**Figure 1E**).

Next, we investigated the difference in the expression of the 21 m6A regulators between the normal and tumor tissues. Fat and muscle tissue samples from the The Genotype-Tissue Expression (GTEx) database were used as adjacent normal tissue samples. Of the 21 regulators, 20 showed significant differences between STS and normal tissues, while ZC3H13 did not (**Figure 1F**). Kaplan–Meier (KM) survival analysis showed significant differences in overall survival between patients with high or low expression of the 21 regulators (**Figure S1B**). The above findings suggested that changes in the expression of m6A regulators may play a crucial role in occurrence and progression of STS.

### Identification of m6A Methylation Modification Patterns

**Figure 2:**
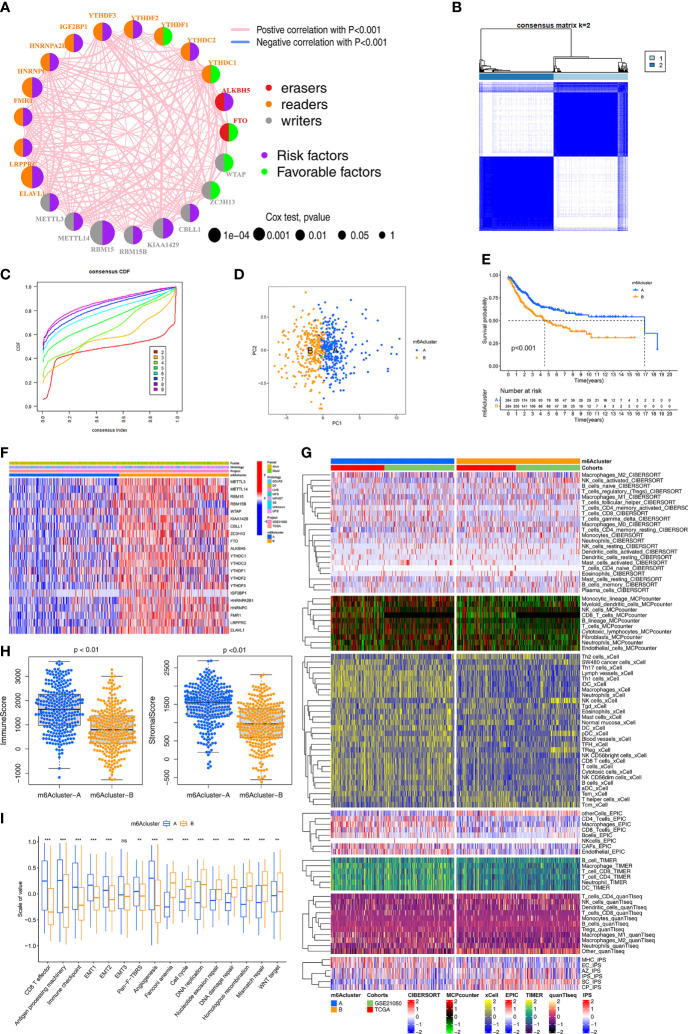
Identification of m6A methylation modification patterns. **(A)** The interaction among m6A regulators in STS. The circle size represented the significance level of P values calculated by Log-rank test, p < 0.001, p < 0.01, p < 0.05 and P < 1, respectively. Favorable factors for overall survival are indicated in green, and risk factors indicated in purple. The lines connecting represent m6A regulators interactions estimated by Spearman correlation analysis. Positive correlation is indicated in pink and negative correlation in blue. **(B)** The clustering heatmap corresponding to the consensus matrix for k=2 obtained by consensus clustering. **(C)** Relative change in area under consensus CDF curve for k=2 to 9. **(D)** Principal component analysis for the transcriptome profiles of two m6A modification subtypes, showing a remarkable difference between different modification patterns. **(E)** Survival analyses for the two m6A modification patterns based on TCGA-SARC and GSE21050 STS cohort including 284 cases in m6Acluster-A, 284 cases in m6Acluster-B. Kaplan-Meier curves with Log-rank p value <0.001 showed a significant survival difference between two m6A modification patterns. **(F)** The expression of 21 m6A regulators between the m6Acluster-A and m6Acluster-B groups and corresponded clinical information also displays in heatmap. **(G)** Heatmap for immune responses based on CIBERSORT, MCPcounter, xCell, EPIC, TIMER, q uanTIseq and iPS algorithms between two m6Aclusters. **(H)** The Immune score and Stromal score from ESTIMATE algorithms of two m6Aclusters were analyzed and plotted. **(I)** The enrichment differences of immune signatures and typical biological processes between the m6Acluster-A and m6Acluster-B groups. The statistical difference was compared through the Kruskal–Wallis test. *P < 0.05; **P < 0.01; ***P < 0.001. ns, no significant.

The crosstalk among the 21 m6A regulators and their prognostic value in STS is comprehensively illustrated in the m6A regulator network (**Figure 2A**). All 21 m6A regulators were positively correlated with each other. Next, consensus clustering was performed using the “ConsensusClusterPlus” R package both TCGA and Gene Expression Omnibus (GEO) cohorts. K = 2 was selected based on the empirical cumulative distribution function (CDF) plots (**Figures 2B, C**). Thus, two m6A modification patterns, designated m6Acluster-A (n=284) and m6Acluster-B (n=284), were identified. STS samples with distinct m6A modification could be completely distinguished (**Figure 2D**). Kaplan–Meier (K-M) survival analysis of the two patterns revealed the clear survival advantage in m6Acluster-A, both TCGA-SARC and GSE21050 cohorts (**Figure 2E**). The expression of the 21 regulators was significantly different between the two patterns (**Figure 2F**). To verify the large difference in survival, the TCGA and GEO cohorts (as the validation dataset) respectively were employed to compare the survival between the two patterns (**Figure S2A**). Importantly, similar results were observed, indicating support for the two patterns identified by the clustering algorithm. Additionally, in the TCGA and GEO cohorts, there were significant differences in the expression of m6A regulators between the two patterns (**Figure S2B**).

Compared to m6Acluster-B, m6Acluster-A had increased enrichment of immune cells (using CIBERSORT, MCP-counter, xCell, EPIC, TIMER, quanTIseq and IPS algorithms), especially regarding anti-tumor immune cells, in the TCGA and GEO cohorts (**Figure 2G**). The ImmuneScore and StromalScore (evaluated by the ESTIMATE method) were compared between the two patterns (**Figure 2H**). The differences in immune cell infiltration between the two patterns were also respectively validated in the TCGA and GEO cohorts, and similar results were observed (**Figure S2C**).

To further explore the biological behaviors in the two patterns, Gene Set Variation Analysis (GSVA) and the “limma” package were used, which led to the identification of 84 differential pathways (**Figure S2D**). Typical biological pathways and immune signatures were compared between the two patterns to explore the potential mechanisms. Pathways related to immunity, metabolism, and stemness (cell cycle, DNA damage repair, DNA replication, and mismatch repair) showed significant differences between the two patterns. In particular, the epithelial-to-mesenchymal transition (EMT) and pan-fibroblast TGF-β response signaling pathways were significantly upregulated in m6Acluster-A, which had strong enrichment of CD8+ T cells, effector antigen processing machinery, and immune checkpoints (**Figure 2I**). DNA damage repair, DNA replication, and Wnt signaling pathways were significantly upregulated in m6Acluster-B. These results demonstrated that m6Acluster-A predominantly featured immune and stromal activation, and m6Acluster-B mainly featured DNA repair. Based on above results, we revealed two m6A modification patterns with distinct characteristics of immunity, metabolism, and stemness, which suggested that m6A modification might regulate immune microenvironment, metabolism processes, and tumor cell stemness to contribute to different behaviors of STS.

### Correlations of the 21 m6A Regulators With Immunity, Metabolic Pathways, and Stemness

To further explore the potential significance of each of the 21 regulators, their correlations with immunity, metabolic pathways, and stemness was analyzed. Regarding immunity, we analyzed the correlations between the expression of the 21 m6A regulators and the infiltration of 28 immune cells (**Figure S3A**).

Regarding metabolic pathways, 40 differential metabolic pathways were identified by the “limma” R package. Subsequently, 18 prognosis-related metabolic pathways were selected by univariate Cox analysis and the randomSurvivalForest algorithm (**Table S2**). The relationship between the error rate and the number of classification trees is shown in **Figure S3B**. After ranking these metabolic pathways by importance according to the out-of-bag error, five metabolic pathways with relative importance >0.5 were considered in the subsequent analysis (**Figure S3C**). Most metabolic pathways were negatively correlated with the 21 m6A regulators (**Figure S3D**).

Regarding stemness, there were significant correlations between the expression of the 21 regulators and the six stemness indices (**Figure S3E**). The highest correlation coefficient was between RBM15 and mRNAsi. Additionally, HNRNPC, YTHDF2, and HNRNPA2B1 were significantly positively correlated with mDNAsi and mRNAsi. K-M survival analysis for the six stemness indices showed a survival advantage in the lower level of stemness index group (**Figure S3F**). Significant correlations between the expression of the 21 regulators with immune microenvironment, metabolic pathways, and stemness levels indicated that these regulators might play important roles in regulation of m6A modification in terms of immunity, metabolism, and stemness for STS.

### Identification of Hub Genes and Immunity/Metabolism/Stemness Subtypes by Co-Expression Network Analysis (WGCNA)

To identify immune/metabolism/stemness phenotype-related genes related to the m6A modification patterns, WGCNA was used to identify biologically meaningful modules corresponding to phenotype-related genes. The 12 phenotypes investigated were ImmuneScore (calculated using the ESTIMATE method), five metabolic pathways (selected in the random survival forest analysis), and the six stemness indices.

**Figure 3:**
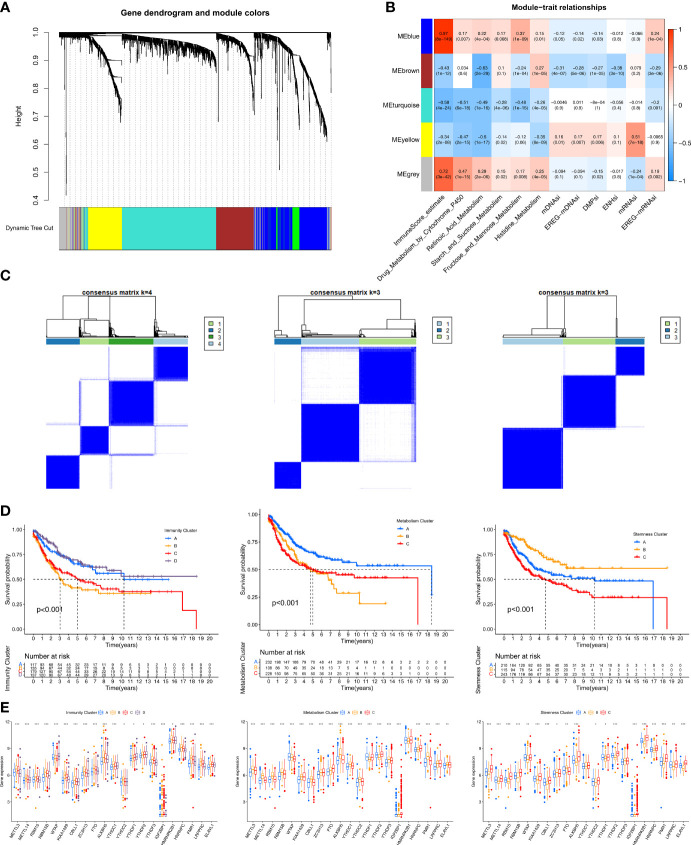
Identification of hub genes and immunity/metabolism/stemness subtypes by WGCNA.**(A)** Hierarchical clustering dendrograms of identified co-expressed genes in modules. The branches of the cluster dendrogram correspond to the different gene modules. Each leaf on the cluster dendrogram corresponds to a gene. Each colored row represents a color-coded module which contains a group of highly connected genes. **(B)** Correlations between the gene modules and clinical traits. The correlation coefficient in each cell represented the correlation between the gene module and the clinical traits. The corresponding P-value and Correlation value are annotated. **(C)** Heatmap corresponding to the consensus matrix for k=4 (left), 3 (middle), 3 (right) obtained by consensus clustering. **(D)** Kaplan–Meier curves using the Log-rank test for immunity (left), metabolism (middle) and stemness (right) clusters respectively. **(E)** The expression of 21 m6A regulators between four immunity (left), metabolism (middle) and stemness (right) clusters. The statistical difference of clusters was compared through the Kruskal–Wallis test. *P < 0.05; **P < 0.01; ***P < 0.001.

First, by comparing the two m6A modification patterns, 2183 differentially expressed genes (DEGs) (|log_2_FC|>0.5 and FDR<0.05) were identified to be used in WGCNA (**Table S3**). In the subsequent WGCNA, there were five modules (merged dynamic) (**Figure 3A**). Of the 12 phenotypes, the three that were most correlated with module genes were selected for further analysis. A heatmap revealed the three key modules (MEblue, MEbrown, and MEyellow for ImmuneScore, Retinoic Acid metabolism pathway, and mRNAsi, respectively) (**Figure 3B**). We further analyzed the correlations of the hub genes in the three modules (**Figure S4A**). Ultimately, we identified 579 immune phenotype (ImmuneScore)-related genes, 326 metabolism phenotype-related (retinoic acid metabolism) genes, and 286 stemness phenotype (mRNAsi)-related genes (**Table S4**).

Three unsupervised consensus clustering analyses were performed based on the immunity/metabolism/stemness phenotype-related genes in the three modules, with the optimal number of clusters being selected based on the corresponding CDF curve (**Figure S4B**). As a result, Immunity groups A–D, Metabolism groups A–C, and Stemness groups A–C, respectively, were defined (**Figure 3C**). Regarding immunity, the K-M survival analysis showed that STS patients in the Immunity A and D groups had better prognoses than those in the Immunity B and C groups (**Figure 3D**). The Immunity A and D groups had massive infiltration of anti-tumor immune cells (CD8+ T cells, macrophages, cytotoxic cells, dendritic cells, and Th1 cells) (**Figure S4C**) and lower expression of m6A regulators (**Figure 3E**). Regarding metabolism, the K-M survival analysis showed that the Metabolism A group had a better prognosis than the Metabolism B and C groups (**Figure 3D**). The three subgroups exhibited different metabolism processes (**Figure S4C**). The Metabolism A group had lower expression of the m6A regulators than the other two groups (**Figure 3E**). Regarding stemness, the K-M survival analysis showed that the Stemness B group had a better prognosis than the Stemness A and C groups (**Figure 3D**). Nevertheless, 15 typical tumor stemness-related biological processes in the three subgroups were comparable (**Figure S4C**). The Stemness B group had lower expression of the m6A regulators than the other two groups (**Figure 3E**).

### Identification of DNA Methylation Subtypes

Using the TCGA-SARC cohort, 531 CpG sites in the 21 m6A regulator genes were identified (**Table S5**). Subsequently, 41 prognosis-related CpG sites were identified by univariate Cox regression. These sites were used to identify DNA methylation subgroups. K = 2 was selected as the most suitable choice based on the consistency of each cluster and the CDF curve (**Figure S5A**). Thus, the DNA methylation site clustering analysis identified two distinct subgroups, designated DNAmethy-Cluster-A and -B (**Figure S5B**). The heatmap shows the differences in the methylation sites in each subgroup (**Figure S5C**), with higher DNA methylation levels in DNAmethy-Cluster-A. The annotated distribution of clinical traits in the heatmap shows that the two subgroups had unique characteristics. K-M survival analysis showed that DNAmethy-Cluster-A had a higher survival rate (**Figure S5D**). Furthermore, six of the m6A regulators (RBM15B, KIAA1429, YTHDF2, HNRNPA2B1, HNRNPC, and ELAVL1) exhibited lower expression in DNAmethy-Cluster-A (**Figure S5E**).

We further explored the differences in immune cell infiltration, metabolic pathways, and stemness between the two DNA methylation subgroups. As expected, DNAmethy-Cluster-A had an immune-activated phenotype characterized by abundant immune cell infiltration (**Figure S5F**). This subgroup was significantly enriched in metabolic pathways, including phenylalanine metabolism, tryptophan metabolism, and nicotinate and nicotinamide metabolic pathways (**Figure S5G**). Furthermore, this group had lower DNA-related stemness index, as shown in the boxplot in **Figure S5H**. These results indicated the key roles of the m6A modification in DNA methylation.

### Identification of m6A Modification Pattern-Related DEGs and Construction of the m6Ascore

STS patients were classified into two m6A modification patterns by consensus clustering based on the expression of 21 m6A regulators. We then examined the potential m6A modification pattern-related gene expression changes between the two patterns, identifying 204 DEGs (log_2_FC>1 and FDR<0.05) (**Table S6**). GO enrichment analysis of these DEGs revealed significant enrichment of T cell mediated immunity, negative regulation of immune response, and positive regulation of cell cycle (**Figure S6A**). The results further demonstrated that the DEGs were characterized by m6A modification, immunity, metabolism pathways, and stemness. The results also confirmed that m6A modification played a key role in the TME. Among the 204 m6A modification pattern-related DEGs, 141 prognosis-related genes were identified by univariate Cox regression (**Table S7**). Subsequently, these 141 genes were subjected to unsupervised consensus clustering analysis and two stable phenotypes were obtained (**Figures S6B, C**). Ultimately, the samples were divided into two distinct m6A gene signature subgroups, designated geneCluster-A and geneCluster-B (**Figure S6D**). The geneCluster-A was associated with better prognosis (**Figure S6E**). There were significant differences in the expression of the 19 m6A regulators between the two subgroups (**Figure S6F**).

Considering the individual heterogeneity and complexity of m6A modification, we quantified the m6A modification pattern of individual STS patients using principal component analysis based on the 141 abovementioned genes. Thus, m6Ascore was defined for each STS patient. We visualized the changes in the attributes of individual patients in different clusters using an alluvial diagram (**Figure S6G**). To assess the prognostic value of m6Ascore, samples were divided into high- and low-m6Ascore using the optimal cutoff (1.46) determined by the “survminer” R package. Survival was higher in the low-m6Ascore group (**Figure S6H**), as verified in the TCGA and GEO cohorts (**Figure S6I**), and the expression levels of the 17 regulators were also significantly different between the two subgroups (**Figure S6J**). Both m6ACluster-A and geneCluster-A had a lower m6Ascore (**Figure S6K**). These results indicate m6Ascore could be used to predict prognosis in STS.

### Correlation Between m6Ascore and Clinicopathological Type

The histological subtypes of STS in the TCGA-SARC and GSE21050 cohorts mainly included undifferentiated pleomorphic sarcoma (UPS; 35.48%), dedifferentiated liposarcoma (DDLPS; 20.11%), desmoid tumor (DT; 0.4%), leiomyosarcoma (LMS; 35.67%), myxofibrosarcoma (MFS; 4.74%), malignant peripheral nerve sheath tumors (MPNST; 1.7%), and synovial sarcoma (SS; 1.9%). K-M survival analysis of these seven subtypes showed that UPS (with a lower m6Ascore) had improved survival, while LMS (with a higher m6Ascore) had poorer survival (**Figure S7A**). The stacked column chart shows the distribution of the histological subtypes in the high- and low-m6Ascore groups (**Figure S7B**). UPS, DDLPS, and MFS patients were mainly in the low-m6Ascore group, while LMS patients were mainly in the high-m6Ascore group (**Figure S7C**). K-M survival analysis of high- and low-m6Ascore subgroups in each histological subtype showed that low-m6Ascore subgroups had a better prognosis, but the difference was only significant for UPS (**Figure S7D**).

### Multiomics Analysis of the Role of m6Ascore

**Figure 4:**
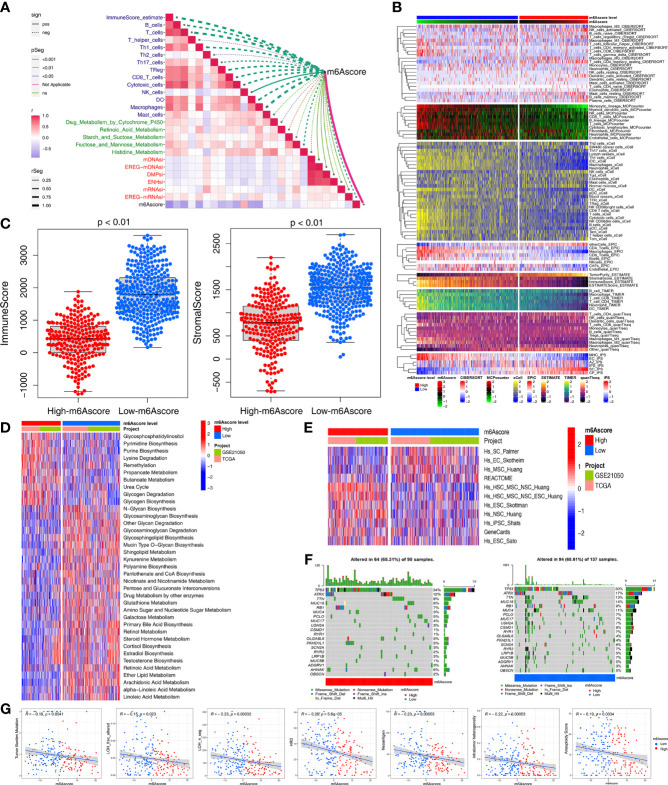
Multiomics analysis of the role of m6Ascore. **(A)** Correlations between m6Ascore and immune cells, metabolic pathways and stemness index, respectively. **(B)** Heatmap for immune responses based on CIBERSORT, MCPcounter, xCell, EPIC, TIMER, quanTIseq and iPS algorithms the low- or high-m6Ascore groups. **(C)** Differences in immuneScore and stromalScore from ESTIMATE algorithms between low- or high-m6Ascore group in the TCGA-SARC and GSE21050 cohort using Kruskal–Wallis test. **(D, E)** GSVA enrichment analysis showing the activation states of metabolic **(D)** and stemness-related **(E)** pathways between high and low m6Ascore groups. The heatmap was used to visualize these pathways, and red represented activated pathways and blue represented inhibited pathways. **(F)** The waterfall plot showing tumor somatic mutation established by those with high m6Ascore (left) and low m6Ascore (right). Each column represented individual patients. The upper barplot showed TMB, the number on the right indicated the mutation frequency in each gene. **(G)** Scatter plots depicting the negative correlation by Spearman correlation analysis between m6Ascore and TMB, neoantigen burden, DNA damage including homologous recombination deficiency (HRD), loss of heterozygosity (LOH; number of segments with LOH events, and fraction of bases with LOH events), intratumor heterogeneity (ITH), and aneuploidy score.

Based on the aforementioned strong associations of the m6A regulators with immunity, metabolism, and stemness in STS patients, we further investigated the correlations of m6Ascore with immunity, metabolism, and stemness. As expected, m6Ascore was significantly correlated with the ImmuneScore and immune cells, including anti-tumor cells (CD8+ T cells, macrophages, Th1 cells, natural killer cells, dendritic cells, and TH17 cells) and pro-tumor immune cells (Th2 cells) (**Figure 4A**). The heatmap of immune cell infiltration (based on CIBERSORT, MCP-counter, xCell, EPIC, TIMER, quanTIseq and IPS algorithms) indicated that the low-m6Ascore group had higher immune cell infiltration, especially regarding anti-tumor-related cells (CD8+ T, dendritic, natural killer, and Th1 cells) (**Figure 4B**). The low-m6Ascore group also had a higher ImmuneScore and StromalScore (calculated using the ESTIMATE method) (**Figure 4C**).

Next, we used the GSVA and “limma” R package to analyze the differences in 114 metabolic pathways between the high- and low-m6Ascore groups, which identified 37 metabolic pathways (**Figure 4D**). Compared to the low-m6Ascore group, the high-m6Ascore group was significantly enriched in the Propanoate metabolism, Lysine degradation, and Glycogen degradation pathways. In addition, there were differences in stemness-related pathways between the two groups (**Figure 4E**).

**Figure 5:**
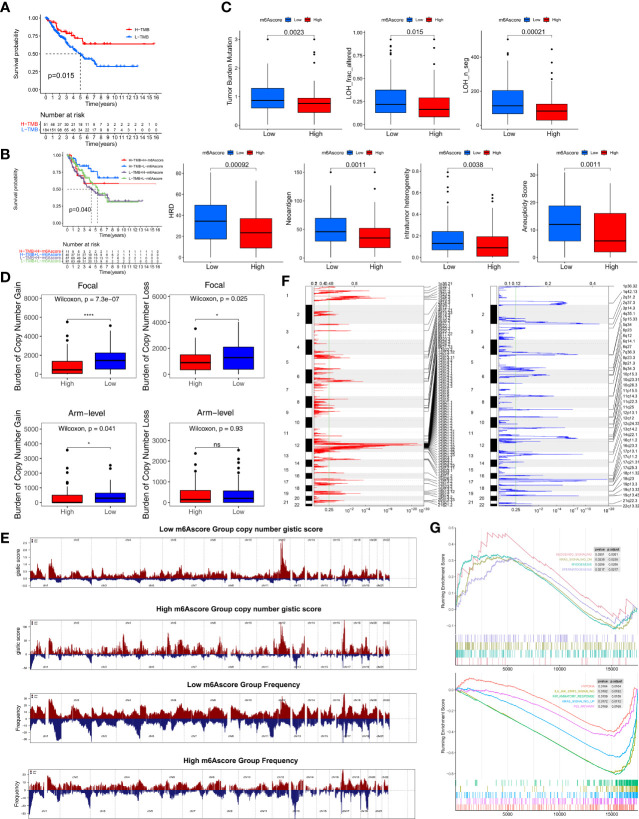
Multiomics analysis of the role of m6Ascore. **(A)** Kaplan-Meier curves depicting survival analyses for low (184 cases) and high (51 cases) TMB patient groups in the TCGA-SARC cohort using Log-rank test. **(B)** Kaplan-Meier curves depicting survival analyses for subgroup patients stratified by both m6Ascore and TMB levels using Log-rank test. **(C)** Differences in the m6Ascore between TMB, neoantigen burden, DNA damage including homologous recombination deficiency (HRD), loss of heterozygosity (LOH; number of segments with LOH events, and fraction of bases with LOH events), intratumor heterogeneity (ITH), and aneuploidy score in the TCGA-SARC cohort. The upper and lower ends of the boxes represented an interquartile range of values. The lines in the boxes represented the median value, and the dots showed outliers. **(D)** Distribution of and focal and broad (arm-level) copy number alterations in the low or high m6Ascore groups. The statistical significance of pairwise comparisons is annotated with symbols in which ns and * represent not significant (P > 0.05) and P ≤ 0.05, respectively. **(E)** Copy number profiles for the low or high m6Ascore groups, with gains in red and losses in blue. Gene segments are placed according to their location on chromosomes, ranging from chromosome 1 to chromosome 22. **(F)** Detailed cytoband with focal amplification (left) and focal deletion (right) in the low-m6Ascore group generated with GISTIC_2.0 software. The q value of each locus is plotted horizontally. **(G)** GSEA plots showing the activated and suppressed gene sets between the high and low m6Ascore groups. Each run was performed with 1,000 permutations. ns, no significant.

We compared the somatic mutations in the TCGA-SARC cohort and found that low-m6Ascore group had a higher tumor mutation rate than the high-m6Ascore group (68.61% versus 65.31%) (**Figure 4F**). Differentially mutated genes between the two groups are displayed as a forest plot (**Figure S7E**). The low m6Ascore group had a markedly higher TMB (**Figure 4G**). Higher TMB was associated with increased survival (**Figure 5A**), and low-m6Ascore combined with higher TMB was also associated with better survival (**Figure 5B**). In addition to TMB, we further studied other immunogenic biomarkers and found that intratumor heterogeneity (LOH), DNA damage including homologous recombination deficiency (HRD), tumor neoantigen burden (TNB), intratumor heterogeneity (ITH), and aneuploidy were significantly negatively correlated with m6Ascore (**Figure 4G**). In summary, the differences in tumor immunogenicity between the high- and low-m6Ascore groups were significant (**Figure 5C**).

We further explored CNV between the two m6Ascore groups. The low-m6Ascore group had a higher focal-level gain (p<0.01) and loss (p=0.02) burden and a higher arm-level gain burden (p=0.04) compared to the high-m6Ascore group (**Figure 5D**). **Figure 5E** shows the distribution of the G-score (based on the frequency and amplitude of the gains and losses) across all chromosomes in the high- and low-m6Ascore groups. Focal amplifications and deletions in various chromosomal regions were detected in both the low- and high-m6Ascore groups (**Figures 5F** and **S7F**). These results show that the low-m6Ascore group had relatively high immunogenicity, while the high-m6Ascore group had relatively low immunogenicity. Moreover, Gene Set Enrichment Analysis (GSEA) showed that the Hedgehog signaling, Myogenesis, and Spermatogenesis pathways were substantially enriched in the high-m6Ascore group, while the Hypoxia, IL6-JAK-STAT3 signaling, Inflammatory response, KRAS signaling, and P53 pathways were enriched in the low-m6Ascore group (**Figure 5G**).

### m6Ascore Predicts Responses to Immunotherapy

**Figure 6:**
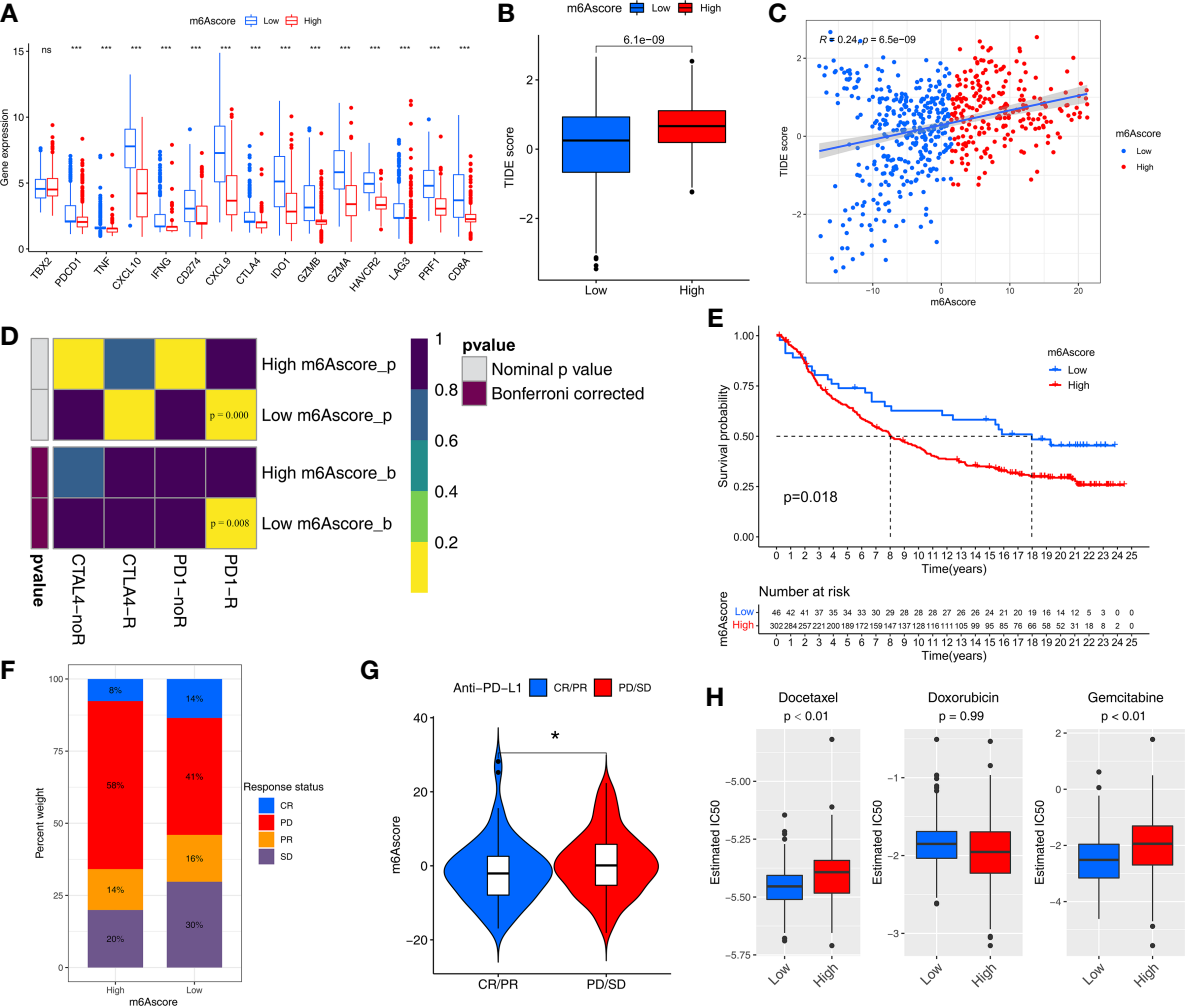
m6Ascore predicts responses to immunotherapy and chemotherapy. **(A)** Differences in the expression of immune checkpoint genes between the low and high m6Ascore groups in the TCGA-SARC and GSE21050 cohort. The statistical difference of clusters was compared through the Kruskal–Wallis test. *P < 0.05; ***P < 0.001. ns, no significant. **(B)** Differences in the TIDE scores between the low and high m6Ascore groups in the TCGA-SARC and GSE21050 cohort. The thick line represents the median value. **(C)** Scatter plots depicting the positive correlation between TIDEscore and m6Ascore in the TCGA-SARC and GSE21050 cohort by the Spearman correlation analysis. The dotted color indicates the low (blue) and high (red) m6Ascore groups. **(D)** Submap analysis manifested that low-m6Ascore groups could be more sensitive to the programmed cell death protein 1 inhibitor (Bonferroni-corrected P = 0.008). **(E)** Kaplan-Meier curves for high and low m6Ascore patient groups in the IMvigor210 cohort. **(F)** The proportion of patients in the IMvigor210 cohort with clinical response in low or highm6Ascore groups. **(G)** Violin plot showing differences in the m6Ascore among patients with different clinical responses in the IMvigor210 cohort using Kruskal–Wallis test. The statistical difference of clusters was compared through the Kruskal–Wallis test. *P < 0.05. **(H)** The box plot of the estimated IC_50_ for Docetaxel, Docetaxel and Gemcitabine are shown between the low and high m6Ascore groups.

We further assessed the ability of m6Ascore to predict the clinical benefit of immunotherapy. The low-m6Ascore group had higher expression of immune checkpoint-related genes (especially regarding PDCD1, PD1, and CTLA4) than the high-m6Ascore group (**Figure 6A**). This suggested that patients with different m6Ascores may have different responses to immune checkpoint inhibitors.

We then used the Tumor Immune Dysfunction and Exclusion (TIDE) algorithm to predict the likelihood of response to immunotherapy, and it demonstrated that the low-m6Ascore group had a lower TIDE score and may therefore be more likely to respond to immunotherapy than the high-m6Ascore group (p<0.01) (**Figure 6B**). m6Ascore was significantly positively correlated with TIDE score (cor=0.24, p<0.01) (**Figure 6C**). We also used Subclass Mapping (SubMap) algorithm to compare the expression profile of the two m6Ascore groups with an independent cohort of 47 melanoma patients treated with immunotherapy. The low-m6Ascore group was more likely to respond to anti–PD-1 antibody treatment (nominal p<0.01, Bonferroni-corrected p<0.01) (**Figure 6D**).

We next explored the prognostic value of the m6Ascore in immune checkpoint inhibitor therapy by classifying patients receiving immune checkpoint inhibitor therapy in the TCGA-SKCM cohort to high or low m6Ascore groups. Patients with high m6Ascores had significantly worse survival than those with lower m6Ascores in TCGA-SKCM cohort (p=0.03) (**Figure S7G**). However, response event outcomes were missing in the clinical information from the TCGA-SKCM cohort, so we further validated the predictive performance of m6Ascore in immunotherapy using an external cohort. The IMvigor210 cohort of 348 anti–PD-L1 antibody (atezolizumab)-treated muscle-invasive bladder cancer patients was used to further validate the value of m6Ascore for predicting the clinical benefit of immunotherapy. Based on our scoring strategy, the m6Ascore of each patient in the IMvigor210 cohort was calculated. The low-m6Ascore group had a significant survival advantage, implying that the m6Ascore reflects sensitivity to immunotherapy (**Figure 6E**). The low-m6Ascore group also mainly included patients who responded to immunotherapy (**Figure 6F**). In addition, the m6Ascore was significantly lower in the complete/partial immunotherapy response group than the non-response group (stable/progressive disease) (**Figure 6G**).

### m6Ascore Could Predict the Sensitivity of Two Chemotherapy Drugs

Chemotherapy regimens generally involve a combination of several anti-cancer drugs. We assessed the response of the low and high-m6Ascore groups to three common chemotherapeutic drugs for STS: docetaxel, doxorubicin, and gemcitabine. We trained a predictive model on a GDSC cell line dataset using ridge regression, with a satisfactory predictive accuracy evaluated by 10-fold cross-validation. The low-m6Ascore group was predicted to be more sensitive to docetaxel (P <0.01) and gemcitabine (P <0.01) (**Figure 6H**).

### Utility of m6Ascore in Pan-Cancer Analysis

To further determine the performance of the m6Ascoring system in various cancer types. The m6Ascores of 10327 samples of 32 cancer types was calculated. Univariate Cox regression indicated that m6Ascore was a favorable factor in ACC, PRAD, MESO, LAML, SKCM, and STAD, and a risk factor in LUAD, PAAD, LGG, KICH, and KIRP (**Figure S8A**). The K-M survival analyses showed that there was a significant difference in overall survival between the high and low m6Ascore groups in the 32 cancer types (p<0.05) (**Figure S9**). Lower m6Ascore improved prognosis in ESCA, COAD, KIRP, BLCA, READ, PAAD, THYM, UCEC, BRCA, KIRC, LUAD, LGG, KICH, CESC, LIHC, and UVM.

The pan-cancer analysis showed that all 32 cancers exhibited a significant correlation between m6Ascore and ImmuneScore (calculated using the ESTIMATE method) (**Figure S8B**). Next, the correlations between m6Ascore and the proportions of 28 immune cells (calculated using the xCell method) were analyzed in the 32 cancer types. The correlation trends in 32 cancer types differed (**Figure S8C**). The proportions of regulatory T cells, M2 macrophages, and Th2 cells (which are all pro-tumor, immunity-suppressing cells) were correlated with m6Ascore in TGCT, PRAD, OV, ACC, GBM, KIRP, LAML, LUAD and LUSC. The proportions of CD8+ T, dendritic, natural killer, and Th1 cells were correlated with m6Ascore in ACC, CESC, LUAD, TGCT and THYM. We further investigated the correlations between m6Ascore and five important metabolic pathways (selected in the random survival forest analysis) in the 32 cancer types and found that 21 cancers were significantly associated with the Retinoic acid metabolism pathway (**Figure S8D**). Additionally, 31 cancers (all except OV) exhibited significant associations between m6Ascore and the six stemness indices (**Figure S8E**), with negative correlations in DLBC and GBM and positive correlations in BRCA, CESC, ESCA, STAD, and LUSC.

TMB, microsatellite instability (MSI), and expression levels of immune checkpoint-related genes can be used to predict the response to immune checkpoint blockade immunotherapy. Of the 32 cancer types, 18 exhibited a significant correlation between m6Ascore and TMB, as shown in radar charts (**Figure S8F**), 13 exhibited a significant correlation between m6Ascore and MSI (**Figure S8G**), and nine (ACC, LIHC, LUAD, LUSC, MESO, OV, PAAD, STAD, and TGCT) exhibited significant correlations between m6Ascores and both TMB and MSI. We further investigated the correlations between m6Ascore and 15 immune checkpoint-related genes in the 32 cancer types, and there was a significant correlation between PD-L1 (CD274) expression and m6Ascore in 29 cancer types (**Figure S8H**), which again confirmed the ability of m6Ascore to predict the clinical benefit of immunotherapy.

## Discussion

In this study, multiomics data and machine learning algorithms were utilized to analyze m6A modification, and we revealed that m6A regulators were involved in the regulation of immunity, metabolism, and stemness in STS, which provides further insights for clinical management, including immunotherapy, chemotherapy and metabolism therapy.

To explore the biological effects of m6A modification in STS, 21 m6A regulators were analyzed based on expression, mutation, and CNV. The high heterogeneity of expression and genomic alterations revealed the pivotal roles of the m6A regulators in STS, which necessitated subsequent analyses. Thereafter, unsupervised clustering was used to identify two m6A modification patterns (m6Acluster-A and -B) with distinct prognoses, distinct characteristics of immunity, metabolism, and stemness in STS. m6Acluster-A had better survival and enrichment of immune-stimulating cells, promoting type I immunity-mediated anti-tumor effects (17, 18). Inversely, elevated Th2 cells reduce type I immunity and facilitate tissue repair (19). Higher B (20), CD8+ T, natural killer, and dendritic cell infiltration and lower Th2 cell infiltration suggested immune activation and contributed to the better prognosis in m6Acluster-A, which was designated the immune-activated phenotype. In contrast, m6Acluster-B, the immune-desert phenotype, had worse prognosis because of the lower immune cell infiltration. Hence, as found in previous studies (21, 22), m6A modification may affect prognosis by regulating the immune microenvironment in STS.

To further investigate the functions of these m6A regulators, we performed GSVA involving typical biological pathways, and we found that the cell cycle, mismatch repair, VEGF signaling, immune-related pathways, metabolic-related pathways, and EMT (23) differed between m6Acluster-A and -B. Higher enrichment scores for the cell cycle and mismatch repair suggested increased cell proliferation in m6Acluster-B, worsening prognosis. Accordingly, we speculated that the m6A regulators also played vital roles in metabolism and stemness in STS, in addition to their roles in immunity. To verify this speculation, the relationships between the m6A regulators and immune cell infiltration, metabolic pathways, and stemness were further explored. In addition to the significant differences of immune cell infiltration between the two m6A modification patterns, the expression levels of the m6A regulators were also correlated with the infiltration of various immune cells. Specifically, most m6A regulators were negatively correlated with dendritic, CD8+ T, and B cells, concurring with the finding that low expression of most m6A regulators was associated with better prognosis. Although the loss of YTHDF1 promotes antigen presentation in DCs (24), YTHDF2 (although not YTHDF1) was negatively correlated with dendritic cell infiltration in STS. Further studies are required to elaborate on the specific mechanisms of each m6A regulator in the immune microenvironment.

Several studies have reported that m6A modification may regulate glycolysis (25, 26), but this was not seen in STS in this study. To investigate the impact of m6A regulators on metabolism in STS, we used the random survival forest algorithm to systematically search for key m6A modification-related prognostic metabolic pathways, and five pathways were identified. Retinoic acid metabolism, drug metabolism by cytochrome P450, and histidine metabolism have been previously reported in STS (27–29). Mounting evidence indicates that metabolism and immunity are closely related to cancer development and progression (30, 31). The significant correlations between m6A regulators and the metabolic pathways suggested that m6A regulators may influence tumor immunity by regulating metabolism, but this requires verification. Consistently, increasing evidence shows that m6A regulators promote cancer stem cell phenotype, EMT, and metastasis in cancers (32, 33). Regarding tumor stemness, in this study, YTHDF2, HNRNPA2B1, HNRNPC, IGF2BP1, and KIAA1429 were positively correlated with tumor stemness, while FTO was negatively correlated with tumor stemness. In addition, our speculation regarding m6A regulators regulating immunity, metabolism, and stemness was supported by GO enrichment analysis of the DEGs between the two m6A modification patterns. Thus, m6A regulators are promising for use as therapeutic targets to influence immunity, metabolism, and stemness, potentially facilitating treatment of STS.

To further investigate genes regulated by m6A modification, the immune/metabolism/stemness phenotype-related genes associated with m6A modification were explored using WGCNA. The three subsequent unsupervised consensus clustering analyses demonstrated that the immune phenotype (ImmuneScore)-, metabolism phenotype (retinoic acid metabolism)-, and stemness phenotype (mRNAsi)-related genes clustered into four, three, and three phenotype clusters, respectively. Each phenotype cluster had unique immunity/metabolism/stemness features and different expression of m6A regulators, contributing to different prognoses. In addition, the immune, metabolism and stemness phenotype-related genes could provide reference for subsequent studies on m6A modification involved in immune, metabolism and stemness in STS. On the other hand, DNA methylation, as a form of epigenetic regulation, can lead to abnormal gene expression, thereby driving oncogenesis (34). Our previous research demonstrated the effect of DNA methylation on the prognosis of STS (35). Therefore, we investigated the regulatory action of DNA methylation on the expression of m6A regulators, with unsupervised clustering leading to the identification of two DNA methylation phenotypes. The improved survival in the higher DNA methylation group could be explained by lower expression of six m6A regulators (RBM15B, KIAA1429, YTHDF2, HNRNPA2B1, HNRNPC, and ELAVL1). Furthermore, the two DNA methylation phenotypes differed in terms of immunity, metabolism, and stemness features. In conclusion, DNA methylation may regulate m6A modification-mediated differences in immunity, metabolism, and stemness.

m6Acluster-A subtype had higher expression of immune checkpoint-related genes than m6Acluster-B, and therefore might be sensitive to immune checkpoint inhibitors. However, the individual-level heterogeneity and complexity of m6A modification cannot be ignored; quantification of m6A modification patterns to distinguish individual differences could guide immunotherapy use in STS. Therefore, we constructed an m6A scoring system designated m6Ascore to quantify the m6A modification patterns in individuals. As expected, m6Ascore had many profound clinical implications. First, it was an outstanding indicator of m6A modification patterns. Second, it overcame the shortcoming of STS histological type, which is a high-performing prognostic factor as indicated by an analysis of 10000 cases (36) but it is often difficult to distinguish different histological types. More specifically, lower m6Ascore was associated with better prognosis in STS among the various histological types, which may be explained by the enriched anti-tumor immune cell infiltration and immune-related pathways in the low m6Ascore group. Third, m6Ascore could help distinguish immunity, metabolism and stemness phenotypes. Fourth, m6Ascore could also predict patient response to immunotherapy and chemotherapy. Patients with higher TMB [an emerging biomarker of immunotherapy responses (37)] in the low m6Ascore group (which had higher sensitivity to immunotherapy) had improved survival. Additionally, immunogenic biomarkers, LOH, HRD, TNB, ITH, and aneuploidy were also significantly negatively correlated with m6Ascore. The key role of m6A modification mediated regulators in modulating DNA repair and genome stability has gradually attracted attention (38). Some m6A methyltransferases can modify and regulate the levels of RNAs involved in DNA damage and repair, which in turn affect genomic instability (39). For example, the m6A methyltransferase METTL3 is activated by ATM-mediated phosphorylation and localized to DNA damage sites, where it promotes HRD repair (40). Previous studies have also shown elevating m6A regulator METTL3 levels could increase the RNA modification of ZBTB4 and decrease levels of ZBTB4 mRNA (41), which in turn increase aneuploidy and genome instability across many frequent human cancers (42). The m6A modification can cause genome instability, which can affect tumor adaptation along with neoantigen production and sensitive to immunotherapy (43, 44). Our study showed the lower m6Ascore and the corresponding higher immunogenicity could contribute to the beneficial effects of immunotherapy, as indicated by a series of machine learning algorithms TIDE (45), SubMap (46), and pairwise comparison analyses (47, 48). Our results indicated that m6A modification, in addition to regulating immunity, metabolism, and stemness, may also be accompanied by changes in immunogenicity. CNV is frequently observed in all kinds of RNA regulatory genes (such as those related to m6A, m5C, m1A, m3C, and m7G), it was reported that CNV of m6A regulator genes is correlated with immune cell infiltration in STS patients (49). Our genome analysis results showed that the low-m6Ascore group, as immune activation group, had more gene mutations and CNV loading burden (including focal and arm-level) than the high-m6Ascore group. The prognostic significance of m6Ascore was also illustrated in other cancer types, and m6Ascore had key roles in immunity, metabolism, and stemness in a pan-cancer analysis in other cancers. Additionally, there were significant correlations between m6Ascore and almost all immune checkpoint-related genes assessed in pan-cancer analysis, further implying that m6A methylation affects immunotherapy sensitivity. The discoveries regarding m6Ascore in the large range of other cancer types are worthy of further study.

This study has several limitations. First, the heterogeneity of samples from the TCGA database combined with the GEO database was disregarded, although the batch-effect correction algorithm was used. Second, there were few STS samples due to the low incidence of STS, and the samples tended to be UPS or DDLPS. Third, there was a lack of clinical cohorts to validate the findings regarding the correlations between m6A modification and the tumor immune landscape and the prognostic value of m6Ascore in STS.

## Data Availability Statement

The datasets presented in this study can be found in online repositories. The names of the repository/repositories and accession number(s) can be found in the article/**Supplementary Material**.

## Ethics Statement 

The patient data in this work were acquired from the publicly available datasets whose informed consent of patients were complete.

## Author Contributions

Z-DH, H-YG, CH and R-XW conceived and designed this study. Z-DH, H-YG and R-XW carried out the analysis procedure. R-XW, Z-DH and L-LL analyzed the results. Z-DH, L-LL, R-XW and Z-ZL contributed analysis tools. Z-DH, R-XW, CH and H-YG participated in the manuscript writing. All the authors reviewed the manuscript.

## Conflict of Interest

The authors declare that the research was conducted in the absence of any commercial or financial relationships that could be construed as a potential conflict of interest.

## Publisher’s Note

All claims expressed in this article are solely those of the authors and do not necessarily represent those of their affiliated organizations, or those of the publisher, the editors and the reviewers. Any product that may be evaluated in this article, or claim that may be made by its manufacturer, is not guaranteed or endorsed by the publisher.

## Supplementary Material

Supplementary Figure S1
**(A)** Overview of study design. **(B)** Survival analyses for the gene expression level of 21 m6A regulators in STS cohort.

Supplementary Figure S2 Different prognosis, infiltration of immune cells and biological processes between two m6A methylation patterns. **(A)** Survival analyses for the two m6A modification patterns in TCGA-SARC (left) and GSE21050 (right) cohort, respectively. Kaplan-Meier curves with Log-rank p value <0.05 showed a significant survival difference between two m6A modification patterns. **(B)** The expression of 21 m6A regulators between m6Acluster-A and m6Acluster-B groups and corresponded clinical information also displays in heatmaps. TCGA-SARC cohort (left) and GSE21050 cohort (right). **(C)** The enrichment differences of 28 immune cell form Xcell algorithm between the m6Acluster-A and m6Acluster-B groups in TCGA-SARC cohort (left) and GSE21050 cohort (right), respectively. The statistical difference of clusters was compared through the Kruskal–Wallis test. *P < 0.05; **P < 0.01; ***P < 0.001. **(D)** GSVA enrichment analysis showing the activation states of biological pathways for distinct m6A modification patterns in TCGA-SARC and GSE21050 cohorts. The heatmap was used to visualize these biological processes, and red represented activated pathways and blue represented inhibited pathways.

Supplementary Figure S3 Correlations of the 21 m6A regulators with immunity, metabolic pathways, and stemness. **(A)** The correlations between 28 immune cell and the expression of each m6A regulator by Pearson analyses. A negative correlation was marked with blue and positive correlation with red. The asterisks represented the statistical p value (*P < 0.05; **P < 0.01; ***P < 0.001). **(B)** Error rate for the data as a function of the classification tree by Random Survival Forest (RSF) algorithm. **(C)** Out-of-bag importance values for the metabolic pathways, the top five metabolic pathways were displayed based on importance value. **(D)** The correlations between 18 metabolic pathways selected by univariate Cox and RSF analyses and the expression of each m6A regulator using Spearman analyses. A negative correlation was marked with blue and positive correlation with red. **(E)** The correlations between six stemness indices and the expression of each m6A regulator using Pearson analyses. A negative correlation was marked with blue and positive correlation with red. **(F)** Kaplan–Meier curves for each type of six stemness indices using the Log-rank test. The high or low level of stemness indices was defined by optimal cut-off using “survminer” R package.

Supplementary Figure S4 Identification of hub genes and immunity/metabolism/stemness subtypes by WGCNA. **(A)** A scatter plot of correlation between blue module eigengene and immunity phenotype (left). A scatter plot of correlation between brown module eigengene and Retinoic Acid metabolism phenotype (middle). A scatter plot of correlation between yellow module eigengene and mRNAsi stemness indices (right). Correlation coefficient and p-value is indicated in the plot. **(B)** Relative change in area under consensus CDF curve for k=2 to 9 in Immunity clustering (left), Metabolism clustering (middle), Stemness clustering (right). **(C)** The abundance of 28 immune cell among Immunity cluster-A, -B, -C, -D groups and corresponded clinical information also displays in heatmap (left). The difference of metabolic pathways among Metabolism cluster-A, -B, -C groups and corresponded clinical information also displays in heatmap (middle). The difference of Stemness pathways among Stemness cluster-A, -B, -C groups and corresponded clinical information also displays in heatmap (right).

Supplementary Figure S5 Identification of distinct DNA methylation subtypes. **(A)** Relative change in area under CDF curve for k=2 to k=9. **(B)** Heatmap corresponding to the consensus matrix for k=2 obtained by using consensus clustering. **(C)** Heatmap of DNA methylation location sites clusters with clinical and molecular pathological parameters. **(D)** Survival analyses for the two DNAmethyclusters based on TCGA-SARC cohort including 258 cases in 147 cases DNAmethycluster-A, 111 cases in DNAmethycluster-B. Kaplan-Meier curves with Log-rank p value 0.001 showed a significant survival difference between two DNA methylation patterns. **(E)** The expression of 21 m6A regulators between DNAmethycluster-A and DNAmethycluster-B. The statistical difference of clusters was compared through the Kruskal–Wallis test. *P < 0.05; **P < 0.01; ***P < 0.001. **(F)** Abundance differences in 28 types of immune cells between DNAmethycluster-A and DNAmethycluster-B in the TCGA-SARC cohort. The statistical difference of clusters was compared through the Kruskal–Wallis test. *P < 0.05; **P < 0.01; ***P < 0.001. **(G)** GSVA enrichment analysis showing the activation states of biological pathways in two distinct DNAmethylationclusters. The heatmap was used to visualize these biological processes, and red represented activated pathways and blue represented inhibited pathways. **(H)** Box plot showing differences in mDNAsi and EREG mDNAsi stemness indices between DNAmethycluster-A and DNAmethycluster-B. The thick line represents the median value. The statistical difference of four groups was compared through the Kruskal–Wallis test.

Supplementary Figure S6 Identification of m6A modification pattern-related DEGs and construction of the m6Ascore. **(A)** GO enrichment analysis of DEGs identified from two m6A modification patterns. The x axis indicates the number of genes within each GO term. **(B)** The cumulative distribution function (CDF) curve is the integral of probability density function using consensus clustering approach. CDF curves of consensus scores based on different subtype number (k = 2 to 9) and the corresponding color are represented. **(C)** The consensus matrix of TCGA-SARC and GSE21050 cohorts using consensus clustering based when k = 2. **(D)** Gene expression heatmap analysis of 141 prognosis-related DEGs between geneCluster-A and geneCluster-B. Heatmap indicates relative gene expression value, with red for high expression and blue for low expression. **(E)** Survival analysis of the two gene cluster subtypes. Kaplan-Meier curves showing the distinct outcomes of STS patients. The P-value was calculated using the log-rank test, by comparing geneCluster-A and geneCluster-B. **(F)** The expression of 21 m6A regulators between geneCluster-A and geneCluster-B. The statistical difference of clusters was compared through the Kruskal–Wallis test. *P < 0.05; **P < 0.01; ***P < 0.001. **(G)** Alluvial diagram showing the changes of m6Acluster, gene cluster, m6Ascore level and survival outcomes. **(H)** Survival analysis of high- and low- m6Ascore groups in TCGA-SARC and GSE21050 cohorts including 568 cases. Kaplan-Meier curves showing the distinct outcomes of STS patients in high- and low- m6Ascore groups. The P-value was calculated using the log-rank test. **(I)** Survival analysis of high- and low- m6Ascore groups in TCGA-SARC cohort including 259 cases (left), GSE21050 cohort including 309 cases (right), respectively. Kaplan-Meier curves showing the distinct outcomes of STS patients in high- and low- m6Ascore groups. The P-value was calculated using the log-rank test. **(J)** The expression of 21 m6A regulators between the high- and low- m6Ascore groups. The statistical difference of clusters was compared through the Kruskal–Wallis test. *P < 0.05; **P < 0.01; ***P < 0.001. **(K)** Differences in m6Ascore between m6Aclusters and geneClusters in TCGA and GSE21050 cohort. (left) The Kruskal-Wallis test was used to compare the statistical m6Ascore difference between m6Acluster-A and m6Acluster-B (P < 0.01). (right) Differences in m6Ascore between geneCluster-A and geneCluster-B using Kruskal-Wallis test.

Supplementary Figure S7 Correlation between m6Ascore and clinicopathological type. **(A)** Survival analyses for patients with each clinical histopathological phenotype in the TCGA-SARC and GSE21050 cohort using Kaplan–Meier method and Log-rank test. **(B)** The proportion of patients with clinical histopathological type in TCGA and GSE21050 cohorts. **(C)** Box plot showing differences in m6AScore among clinical histopathological type in the TCGA-SARC and GSE21050 cohorts. The statistical difference of clusters was compared through the Kruskal–Wallis test. *P < 0.05; **P < 0.01; ***P < 0.001. **(D)** Survival analyses for each histology type including in the TCGA and GEO cohort using Kaplan–Meier method and Log-rank test. R package “survminer” was used to determine the optimal cutpoint for the levels of m6Ascore. **(E)** Forest plot of the differentially mutated genes between high and low m6Ascore subgroups. The statistical difference of the two groups was compared through the Fisher exact test. *P < 0.05; **P < 0.01; ***P < 0.001. **(F)** Detailed cytoband with focal amplification (left) and focal deletion (right) in the high-m6Ascore group generated with GISTIC_2.0 software. The q value of each locus is plotted horizontally. **(G)** Kaplan–Meier curves of overall survival of patients treated with lpilimumab in TCGA-SKCM cohort.

Supplementary Figure S8 The utility of m6Ascore in pan-cancer. **(A)** The overall survival analyses for the m6Ascore in TCGA cancer types using a univariate Cox regression model. Hazard ratio > 1 represented risk factors for survival and hazard ratio < 1 represented protective factors for survival. **(B)** Radar chart of the correlation between m6Ascore and immune score from ESTIMATE. The dots in the radar chart represent the R-value of correlation: R > 0, positive correlation; and R < 0, negative correlation. **(C)** Correlations between the m6Ascore and immune cell for each cancer type (Pearson test). The upper part of each grid showed the P-value, and the bottom part showed the correlation coefficient. The asterisks represented the statistical P-value. (Pearson test, *P < 0.05; **P < 0.01; ***P < 0.001). **(D)** Correlations between the m6Ascore and metabolic pathways for each cancer type (Pearson test). The asterisks represented the statistical p value (*P < 0.05; **P < 0.01; ***P < 0.001). **(E)** Correlation between the m6Ascore and six stemness indices for each cancer type (Pearson test). The asterisks represented the statistical p value (*P < 0.05; **P < 0.01; ***P < 0.001). **(F–G)** Radar chart of the correlation between m6Ascore and tumor mutation burden **(F)**, microsatellite instability **(G)**. **(H)** Correlation between the m6Ascore and immune checkpoint–related genes for each cancer type. The asterisks represented the statistical p value (*P < 0.05; **P < 0.01; ***P < 0.001).

Supplementary Figure S9 Survival analyses for the level of m6Ascore in 32 cancer types from TCGA cohorts.

Supplementary Table S1 The numbers of samples and the clinical baseline and endpoint data of each STS sample in TCGA-SARC and GSE21050 cohorts.

Supplementary Table S2 Prognosis-related metabolic pathways selected by univariate Cox analysis, multivariate Cox analysis and the randomSurvivalForest algorithm.

Supplementary Table S3 2183 DEGs (|log_2_FC|>0.5 and FDR<0.05) by comparing the two m6A modification patterns.

Supplementary Table S4 579 immune phenotype (ImmuneScore)-related genes, 326 metabolism phenotype-related (retinoic acid metabolism) genes, and 286 stemness phenotype (mRNAsi)-related genes.

Supplementary Table S5 The results of univariate and multivariate Cox analysis for 531 CpG sites in the 21 m6A regulator genes.

Supplementary Table S6 204 DEGs (log_2_FC>1 and FDR<0.05) by comparing the two m6A modification patterns.

Supplementary Table S7 Identification of prognosis-related genes were identified by univariate and multivariate Cox regression.
