# Supplementary figures and images for: m6A Modification Patterns With Distinct Immunity, Metabolism, and Stemness Characteristics in Soft Tissue Sarcoma
Source: Front Immunol. 2021 Dec 24;12:765723. doi: 10.3389/fimmu.2021.765723 (PMC8739240; doi:10.3389/fimmu.2021.765723)

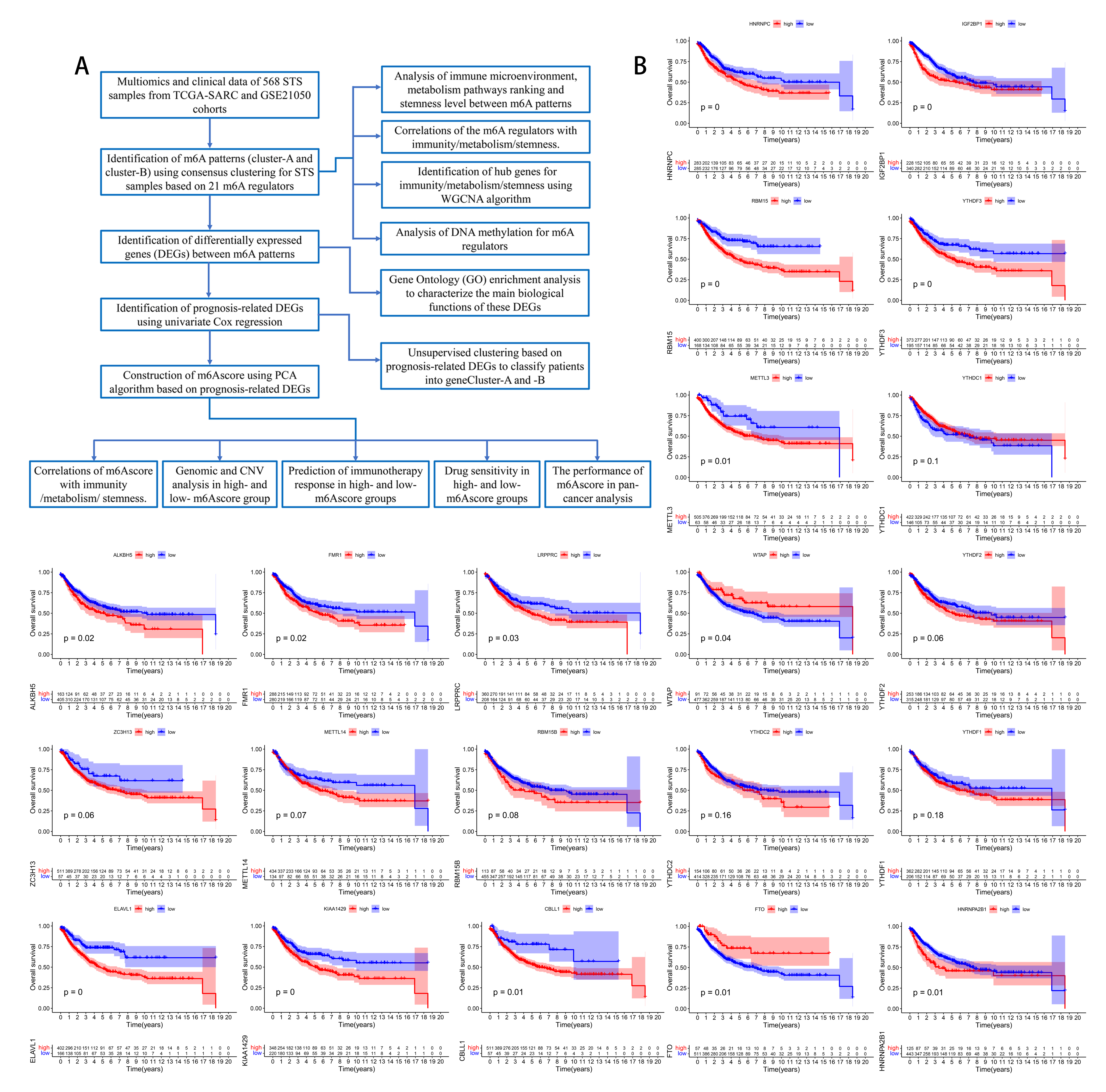

Supplement: Supplementary Figure S1 — (A) Overview of study design. (B) Survival analyses for the gene expression level of 21 m6A regulators in STS cohort. [file Image_1.tif]

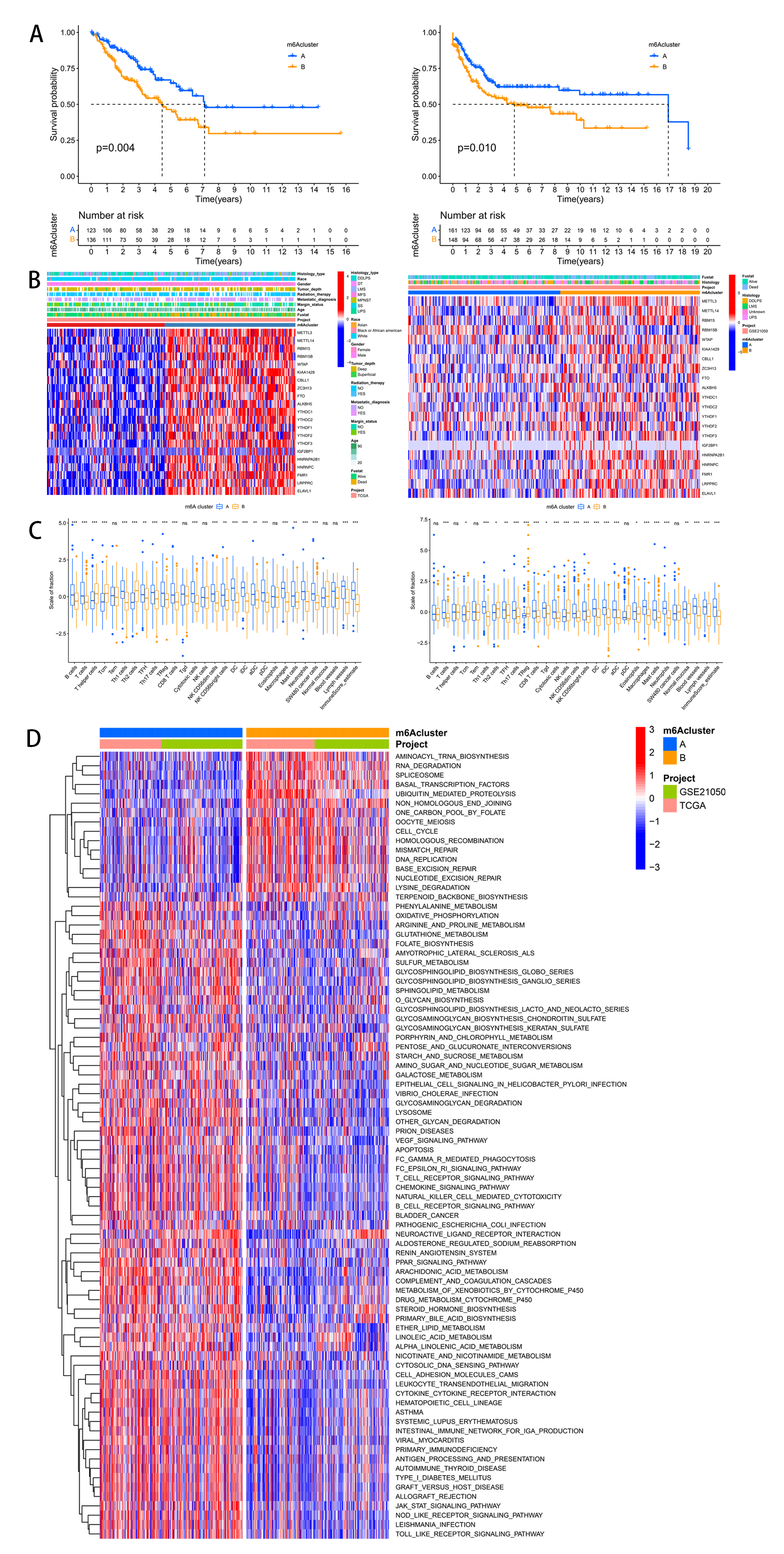

Supplement: Supplementary Figure S2 — Different prognosis, infiltration of immune cells and biological processes between two m6A methylation patterns. (A) Survival analyses for the two m6A modification patterns in TCGA-SARC (left) and GSE21050 (right) cohort, respectively. Kaplan-Meier curves with Log-rank p value <0.05 showed a significant survival difference between two m6A modification patterns. (B) The expression of 21 m6A regulators between m6Acluster-A and m6Acluster-B groups and corresponded clinical information also displays in heatmaps. TCGA-SARC cohort (left) and GSE21050 cohort (right). (C) The enrichment differences of 28 immune cell form Xcell algorithm between the m6Acluster-A and m6Acluster-B groups in TCGA-SARC cohort (left) and GSE21050 cohort (right), respectively. The statistical difference of clusters was compared through the Kruskal–Wallis test. *P < 0.05; **P < 0.01; ***P < 0.001. (D) GSVA enrichment analysis showing the activation states of biological pathways for distinct m6A modification patterns in TCGA-SARC and GSE21050 cohorts. The heatmap was used to visualize these biological processes, and red represented activated pathways and blue represented inhibited pathways. [file Image_2.tif]

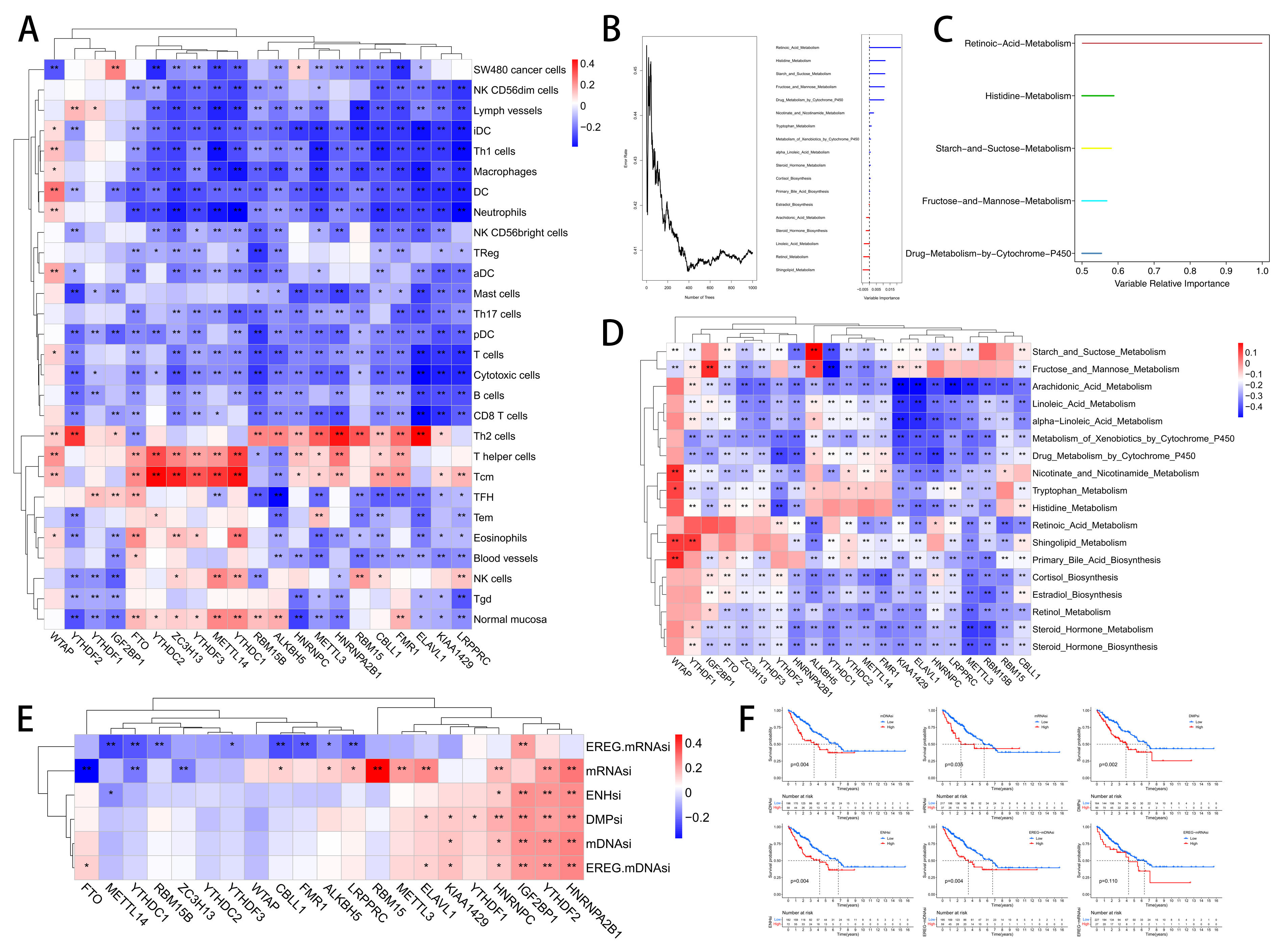

Supplement: Supplementary Figure S3 — Correlations of the 21 m6A regulators with immunity, metabolic pathways, and stemness. (A) The correlations between 28 immune cell and the expression of each m6A regulator by Pearson analyses. A negative correlation was marked with blue and positive correlation with red. The asterisks represented the statistical p value (*P < 0.05; **P < 0.01; ***P < 0.001). (B) Error rate for the data as a function of the classification tree by Random Survival Forest (RSF) algorithm. (C) Out-of-bag importance values for the metabolic pathways, the top five metabolic pathways were displayed based on importance value. (D) The correlations between 18 metabolic pathways selected by univariate Cox and RSF analyses and the expression of each m6A regulator using Spearman analyses. A negative correlation was marked with blue and positive correlation with red. (E) The correlations between six stemness indices and the expression of each m6A regulator using Pearson analyses. A negative correlation was marked with blue and positive correlation with red. (F) Kaplan–Meier curves for each type of six stemness indices using the Log-rank test. The high or low level of stemness indices was defined by optimal cut-off using “survminer” R package. [file Image_3.tif]

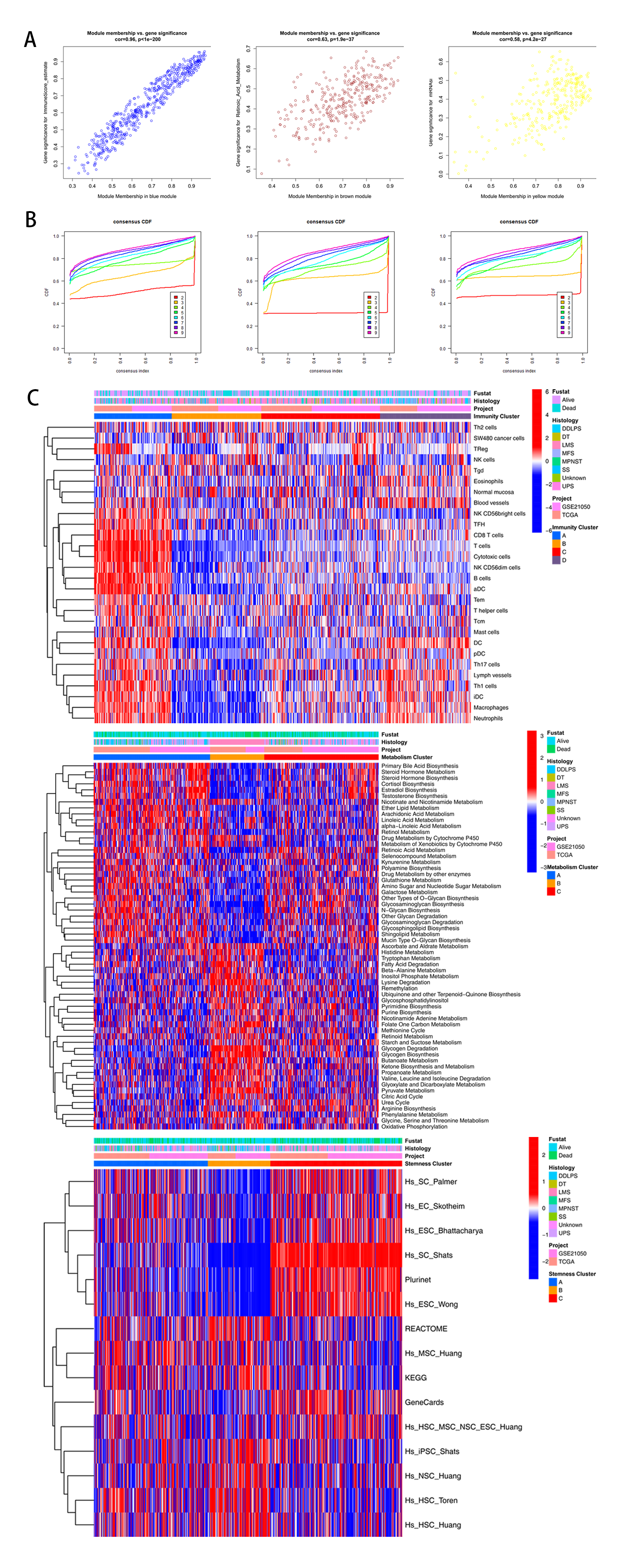

Supplement: Supplementary Figure S4 — Identification of hub genes and immunity/metabolism/stemness subtypes by WGCNA. (A) A scatter plot of correlation between blue module eigengene and immunity phenotype (left). A scatter plot of correlation between brown module eigengene and Retinoic Acid metabolism phenotype (middle). A scatter plot of correlation between yellow module eigengene and mRNAsi stemness indices (right). Correlation coefficient and p-value is indicated in the plot. (B) Relative change in area under consensus CDF curve for k=2 to 9 in Immunity clustering (left), Metabolism clustering (middle), Stemness clustering (right). (C) The abundance of 28 immune cell among Immunity cluster-A, -B, -C, -D groups and corresponded clinical information also displays in heatmap (left). The difference of metabolic pathways among Metabolism cluster-A, -B, -C groups and corresponded clinical information also displays in heatmap (middle). The difference of Stemness pathways among Stemness cluster-A, -B, -C groups and corresponded clinical information also displays in heatmap (right). [file Image_4.tif]

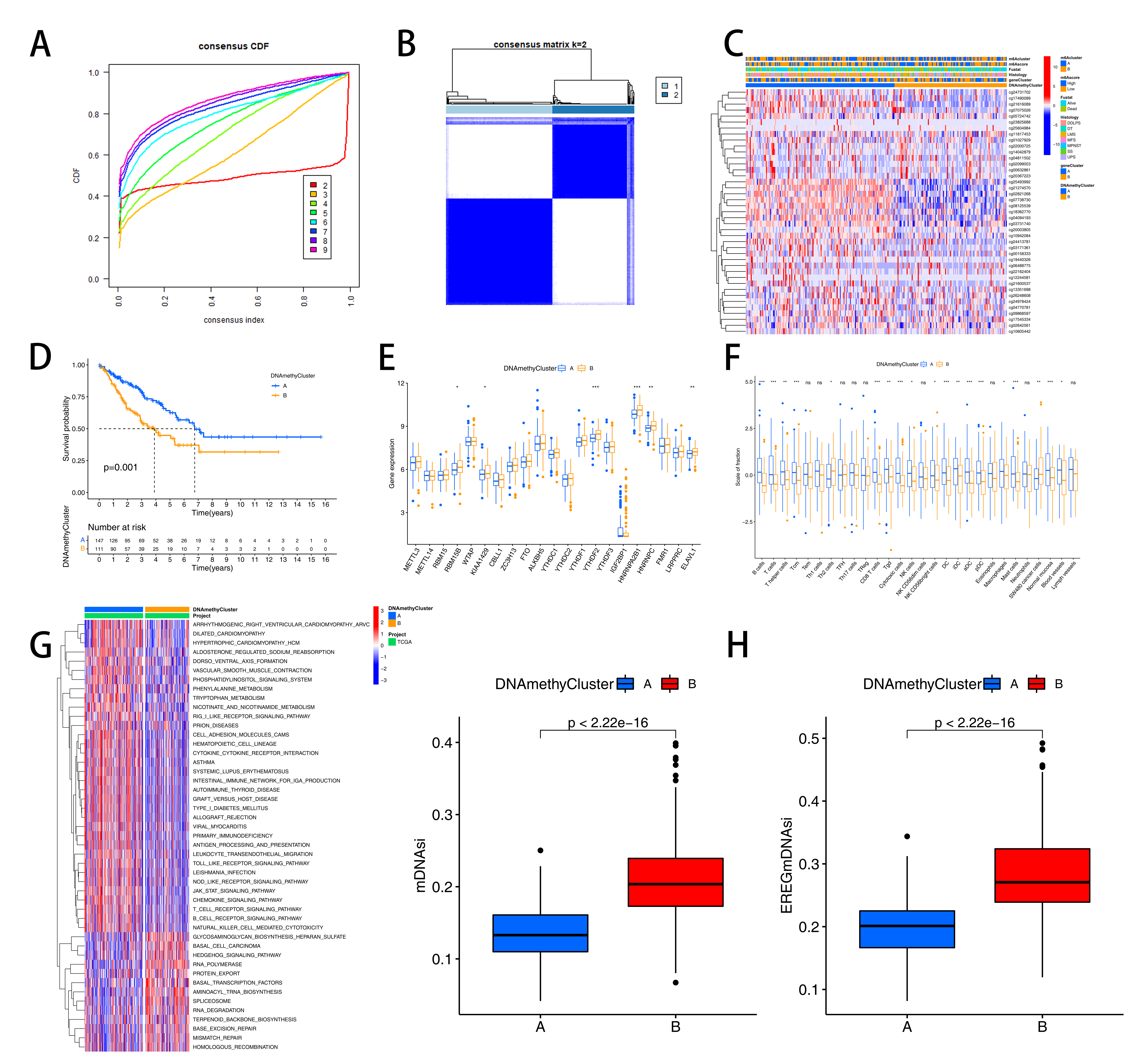

Supplement: Supplementary Figure S5 — Identification of distinct DNA methylation subtypes. (A) Relative change in area under CDF curve for k=2 to k=9. (B) Heatmap corresponding to the consensus matrix for k=2 obtained by using consensus clustering. (C) Heatmap of DNA methylation location sites clusters with clinical and molecular pathological parameters. (D) Survival analyses for the two DNAmethyclusters based on TCGA-SARC cohort including 258 cases in 147 cases DNAmethycluster-A, 111 cases in DNAmethycluster-B. Kaplan-Meier curves with Log-rank p value 0.001 showed a significant survival difference between two DNA methylation patterns. (E) The expression of 21 m6A regulators between DNAmethycluster-A and DNAmethycluster-B. The statistical difference of clusters was compared through the Kruskal–Wallis test. *P < 0.05; **P < 0.01; ***P < 0.001. (F) Abundance differences in 28 types of immune cells between DNAmethycluster-A and DNAmethycluster-B in the TCGA-SARC cohort. The statistical difference of clusters was compared through the Kruskal–Wallis test. *P < 0.05; **P < 0.01; ***P < 0.001. (G) GSVA enrichment analysis showing the activation states of biological pathways in two distinct DNAmethylationclusters. The heatmap was used to visualize these biological processes, and red represented activated pathways and blue represented inhibited pathways. (H) Box plot showing differences in mDNAsi and EREG mDNAsi stemness indices between DNAmethycluster-A and DNAmethycluster-B. The thick line represents the median value. The statistical difference of four groups was compared through the Kruskal–Wallis test. [file Image_5.tif]

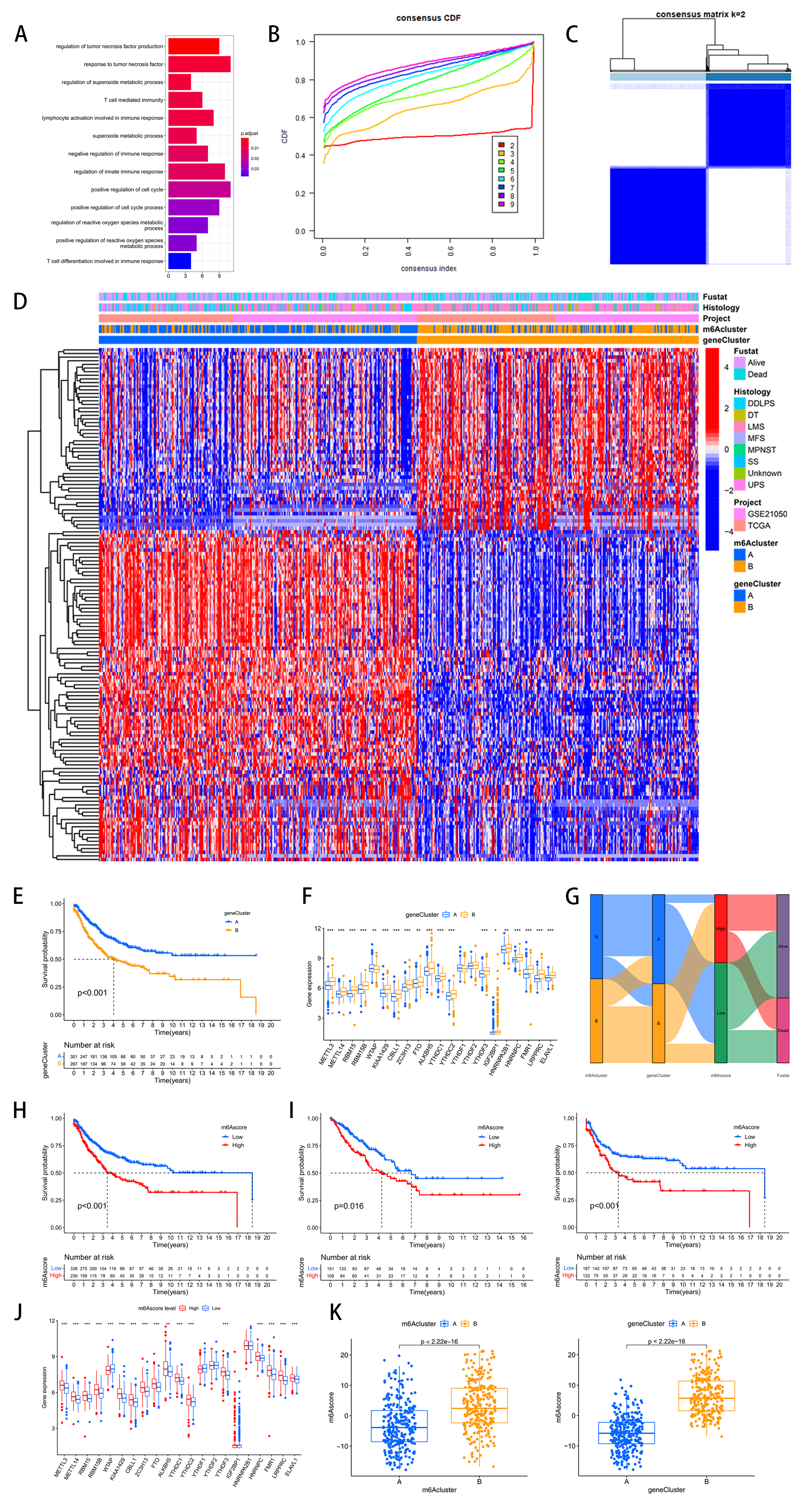

Supplement: Supplementary Figure S6 — Identification of m6A modification pattern-related DEGs and construction of the m6Ascore. (A) GO enrichment analysis of DEGs identified from two m6A modification patterns. The x axis indicates the number of genes within each GO term. (B) The cumulative distribution function (CDF) curve is the integral of probability density function using consensus clustering approach. CDF curves of consensus scores based on different subtype number (k = 2 to 9) and the corresponding color are represented. (C) The consensus matrix of TCGA-SARC and GSE21050 cohorts using consensus clustering based when k = 2. (D) Gene expression heatmap analysis of 141 prognosis-related DEGs between geneCluster-A and geneCluster-B. Heatmap indicates relative gene expression value, with red for high expression and blue for low expression. (E) Survival analysis of the two gene cluster subtypes. Kaplan-Meier curves showing the distinct outcomes of STS patients. The P-value was calculated using the log-rank test, by comparing geneCluster-A and geneCluster-B. (F) The expression of 21 m6A regulators between geneCluster-A and geneCluster-B. The statistical difference of clusters was compared through the Kruskal–Wallis test. *P < 0.05; **P < 0.01; ***P < 0.001. (G) Alluvial diagram showing the changes of m6Acluster, gene cluster, m6Ascore level and survival outcomes. (H) Survival analysis of high- and low- m6Ascore groups in TCGA-SARC and GSE21050 cohorts including 568 cases. Kaplan-Meier curves showing the distinct outcomes of STS patients in high- and low- m6Ascore groups. The P-value was calculated using the log-rank test. (I) Survival analysis of high- and low- m6Ascore groups in TCGA-SARC cohort including 259 cases (left), GSE21050 cohort including 309 cases (right), respectively. Kaplan-Meier curves showing the distinct outcomes of STS patients in high- and low- m6Ascore groups. The P-value was calculated using the log-rank test. (J) The expression of 21 m6A regulators between the high- and low- m6Asco [file Image_6.tif]

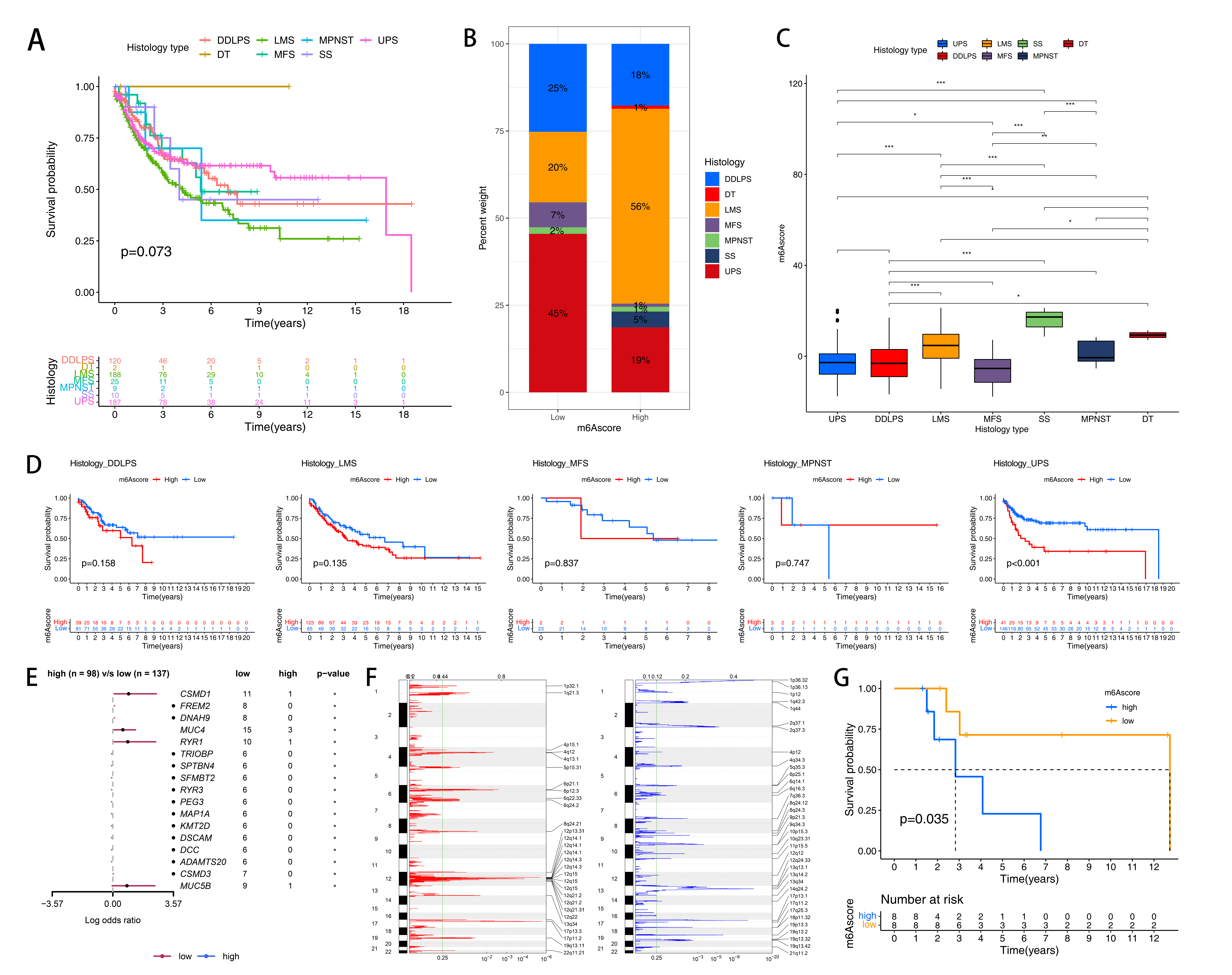

Supplement: Supplementary Figure S7 — Correlation between m6Ascore and clinicopathological type. (A) Survival analyses for patients with each clinical histopathological phenotype in the TCGA-SARC and GSE21050 cohort using Kaplan–Meier method and Log-rank test. (B) The proportion of patients with clinical histopathological type in TCGA and GSE21050 cohorts. (C) Box plot showing differences in m6AScore among clinical histopathological type in the TCGA-SARC and GSE21050 cohorts. The statistical difference of clusters was compared through the Kruskal–Wallis test. *P < 0.05; **P < 0.01; ***P < 0.001. (D) Survival analyses for each histology type including in the TCGA and GEO cohort using Kaplan–Meier method and Log-rank test. R package “survminer” was used to determine the optimal cutpoint for the levels of m6Ascore. (E) Forest plot of the differentially mutated genes between high and low m6Ascore subgroups. The statistical difference of the two groups was compared through the Fisher exact test. *P < 0.05; **P < 0.01; ***P < 0.001. (F) Detailed cytoband with focal amplification (left) and focal deletion (right) in the high-m6Ascore group generated with GISTIC_2.0 software. The q value of each locus is plotted horizontally. (G) Kaplan–Meier curves of overall survival of patients treated with lpilimumab in TCGA-SKCM cohort. [file Image_7.tif]

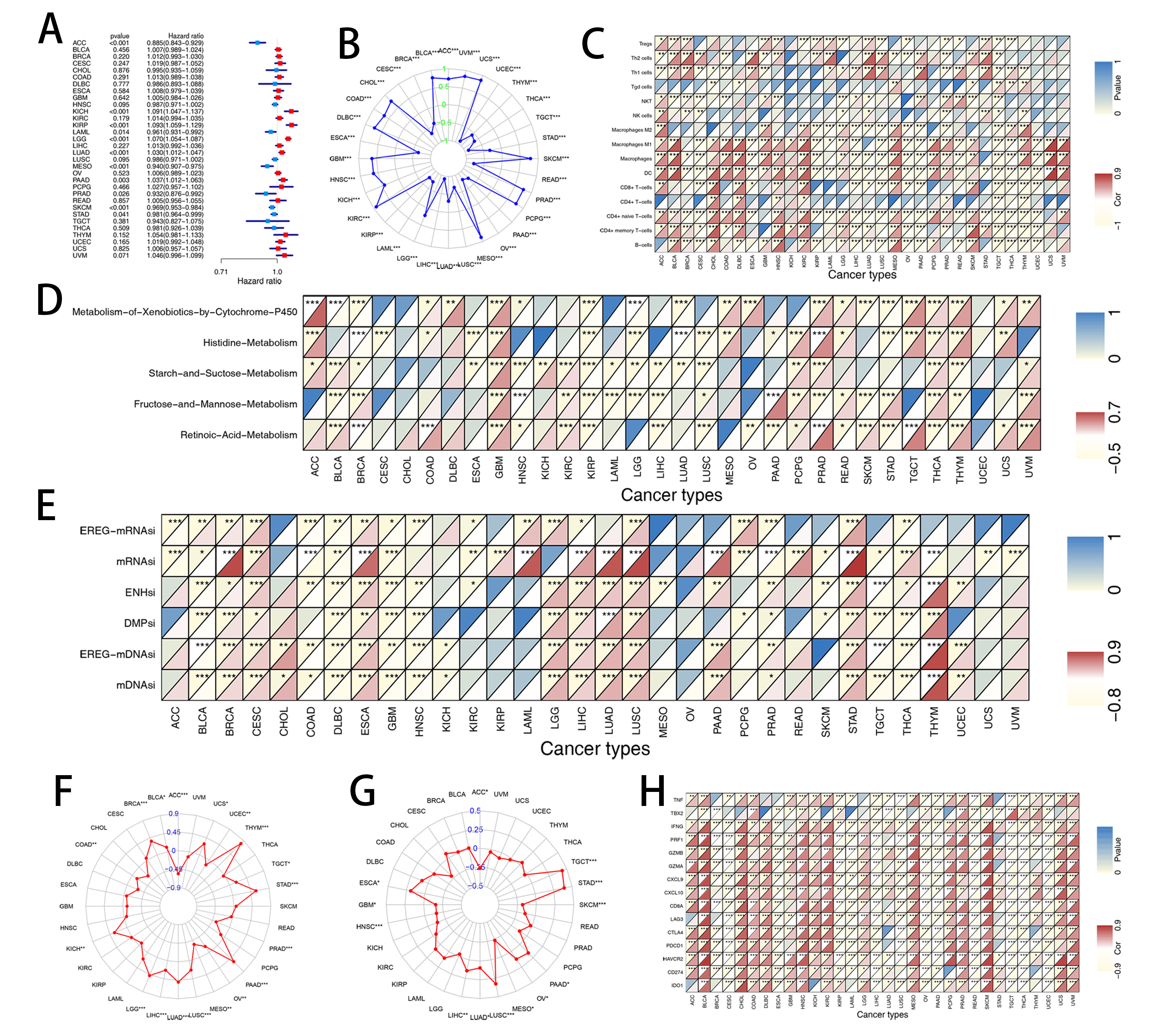

Supplement: Supplementary Figure S8 — The utility of m6Ascore in pan-cancer. (A) The overall survival analyses for the m6Ascore in TCGA cancer types using a univariate Cox regression model. Hazard ratio > 1 represented risk factors for survival and hazard ratio < 1 represented protective factors for survival. (B) Radar chart of the correlation between m6Ascore and immune score from ESTIMATE. The dots in the radar chart represent the R-value of correlation: R > 0, positive correlation; and R < 0, negative correlation. (C) Correlations between the m6Ascore and immune cell for each cancer type (Pearson test). The upper part of each grid showed the P-value, and the bottom part showed the correlation coefficient. The asterisks represented the statistical P-value. (Pearson test, *P < 0.05; **P < 0.01; ***P < 0.001). (D) Correlations between the m6Ascore and metabolic pathways for each cancer type (Pearson test). The asterisks represented the statistical p value (*P < 0.05; **P < 0.01; ***P < 0.001). (E) Correlation between the m6Ascore and six stemness indices for each cancer type (Pearson test). The asterisks represented the statistical p value (*P < 0.05; **P < 0.01; ***P < 0.001). (F–G) Radar chart of the correlation between m6Ascore and tumor mutation burden (F), microsatellite instability (G). (H) Correlation between the m6Ascore and immune checkpoint–related genes for each cancer type. The asterisks represented the statistical p value (*P < 0.05; **P < 0.01; ***P < 0.001). [file Image_8.tif]

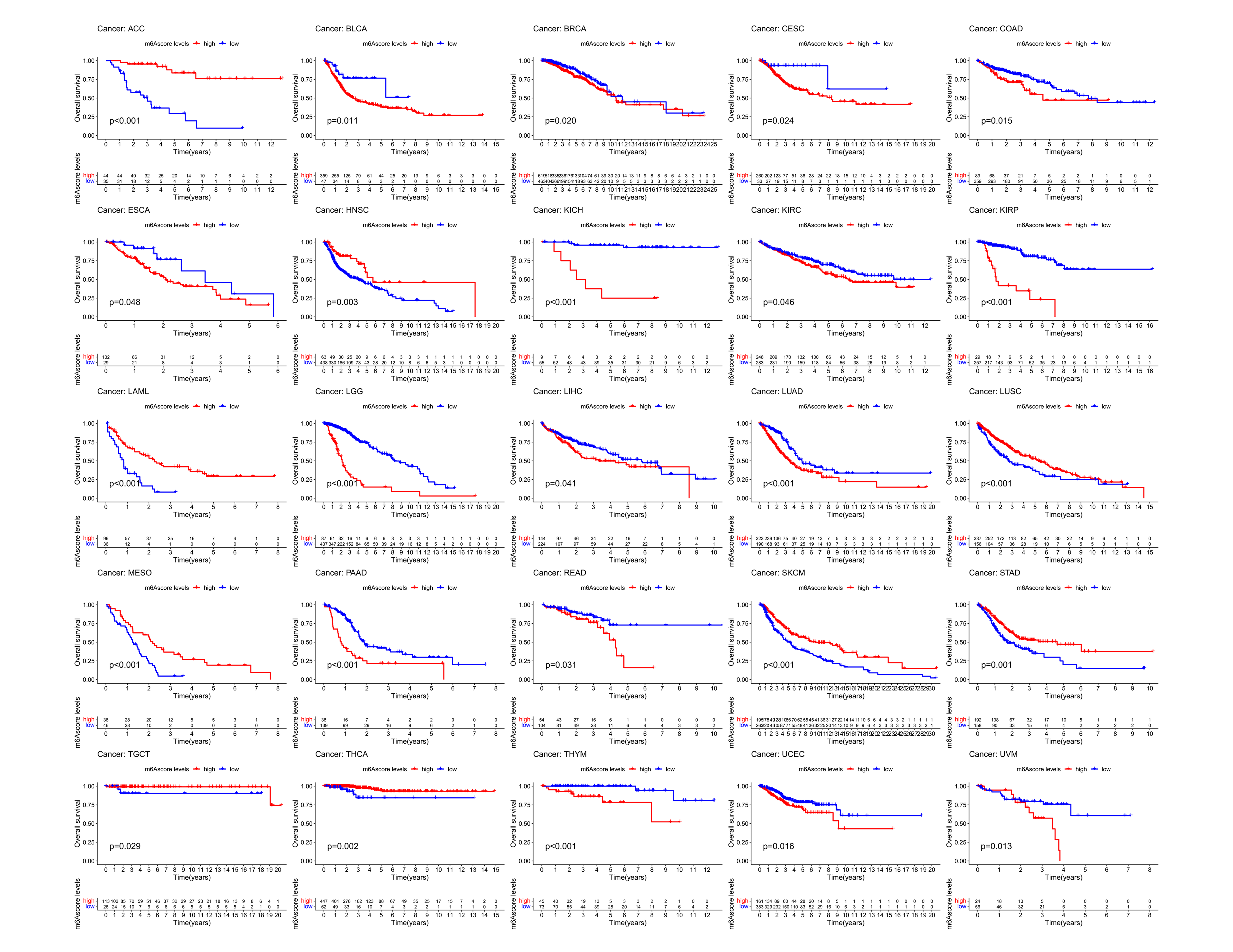

Supplement: Supplementary Figure S9 — Survival analyses for the level of m6Ascore in 32 cancer types from TCGA cohorts. [file Image_9.tif]
