# Supplementary material for: m6A Modification Patterns With Distinct Immunity, Metabolism, and Stemness Characteristics in Soft Tissue Sarcoma
Source: Front Immunol. 2021 Dec 24;12:765723. doi: 10.3389/fimmu.2021.765723 (PMC8739240; doi:10.3389/fimmu.2021.765723)
Supplement: Supplementary file 11 [file DataSheet_2.docx]

**Materials and Methods**

**STS datasets and preprocessing**

Level 4 gene expression data (Fragments Per Kilobase of transcript per Million mapped reads [FPKM] normalized) for STS cases in TCGA were downloaded from the UCSC Xena browser (GDC hub: https://gdc.xenahubs.net). GSE21050 microarray data on STS cases were downloaded from the GEO database (https://www.ncbi.nlm.nih.gov/geo/). Gene expression data for muscle and fat tissues samples (n=911) were downloaded from the The Genotype-Tissue Expression (GTEx) database for use as matched controls. The TCGA gene expression data were transformed into transcripts per kilobase million (TPM) values. GTEx gene expression data were also log2(x+1) transformed to allow comparisons with the TCGA data. Batch effects from non-biological technical biases were corrected using the “ComBat” algorithm in the “sva” R package.

**Clinical and mutation data and preprocessing**

We obtained clinical data corresponding to TCGA-SARC and GSE21050 cohorts, including overall survival time and histological type, from the UCSC Xena browser and GEO database. The numbers of samples and the clinical baseline and endpoint data of each STS sample are summarized in **Table S1**. Genomic mutation data (somatic mutation and copy number variation [CNV]) of TCGA-SARC were also obtained from the UCSC Xena database. TMB was calculated as the number of non-synonymous somatic, coding, base substitution, and indel mutations per mega-base (Mb). The “maftools” R package ([1](#_ENREF_1)) was used to visualize the mutation landscape of the STS samples. The “Rcircos” R package ([2](#_ENREF_2)) was used to plot the data on the CNV landscape of 21 m6A regulators across the 23 pairs of human chromosomes. For CNV analysis, we used GISTIC_2.0 ([3](#_ENREF_3)) to identify significant amplifications and deletions. The CNV gain or loss burden was calculated as the total number of genes with CNV at the focal and arm levels using GenePattern (https://cloud.genepattern.org).

**Unsupervised clustering of 21 m6A regulators**

Unsupervised clustering analysis was used to identify patient subgroups with distinct m6A modification patterns based on the expression of 21 m6A regulators (8 “writers”: CBLL1, KIAA1429, METTL14, METTL3, RBM15, RBM15B, WTAP, ZC3H13; 11 “readers”: ELAVL1, FMR1, HNRNPA2B1, HNRNPC, IGF2BP1, LRPPRC, YTHDC1, YTHDC2, YTHDF1, YTHDF2, YTHDF3; and 2 “erasers”: ALKBH5 and FTO), which were obtained from a previous study ([4](#_ENREF_4)). The cases were classed by k-means, with k from 2 to 9 using the “ConsensusClusterPlus” R package ([5](#_ENREF_5)), with 1000 repetitions to ensure classification stability. The optimal number of clusters was defined by computing the dispersion of the resulting consensus clustering matrix and CDF curve.

**Proportions of infiltrating immune cells**

To quantify the proportions of immune cells in the STS samples, we utilized the “immunedeconv” R package ([6](#_ENREF_6)), which integrates multiple immune cell infiltration estimation algorithms, including TIMER, xCell, MCP-counter, CIBERSORT, EPIC, quanTIseq and IPS. In addition, the “ESTIMATE” R package ([7](#_ENREF_7)) was used to estimate the TME components, in terms of the ImmuneScore, StromalScore and ESTIMATEScore, of each sample.

**Gene set variation analysis (GSVA)**

GSVA was performed using the “gsva” R package ([8](#_ENREF_8)) to evaluate pathway activity in the two m6A modification patterns. We obtained well-defined biological signatures from the Kyoto Encyclopedia of Genes and Genomes (KEGG) gene set, the Hallmark gene set v7.1 from the MSigDB database (https://www.gsea-msigdb.org/gsea/msigdb/), 114 metabolism-related gene signatures from a previous study ([9](#_ENREF_9)), and a typical tumor-related biological process gene set from the “IMvigor210CoreBiologies” R package ([10](#_ENREF_10)). Finally, GSVA was performed to calculate patient-specific GSVA scores that quantified the pathway activity.

**Gene Ontology (GO) annotation and GSEA**

Gene annotation enrichment using the “clusterProfiler” R package ([11](#_ENREF_11)), with q<0.05 as the cutoff. We also identified terms and pathways that were up- and downregulated using GSEA ([12](#_ENREF_12)). Pathway activity was explored using the background collection of gene sets from the MSigDB and KEGG databases. Upregulated pathways were defined based on enrichment score (ES)>0, while downregulated pathways were defined based on ES<0. Enrichment p values were based on 10,000 permutations and subsequently adjusted for multiple testing using the Benjamini–Hochberg procedure to control the false discovery rate (FDR).

**Correlations of m6A regulators with immune cell infiltration, metabolic pathways, and stemness index**

The infiltration of 28 immune cells in each STS sample was calculated using the xCell algorithm ([13](#_ENREF_13)). Regarding metabolic pathways, GSVA was used to calculate scores for 114 metabolic pathways identified in a previous study ([9](#_ENREF_9)) for each STS sample. To select the prognosis-related metabolic pathways, univariate Cox regression was performed for each metabolic pathway score and survival data using the coxph function in the “survival” R package ([14](#_ENREF_14)), with p<0.05 as the cutoff. We further used the randomSurvivalForest algorithm ([15](#_ENREF_15)) to rank the importance of the resultant prognosis-related metabolic pathways (nrep = 100, which indicates that the number of iterations in the Monte Carlo simulation was 100; nstep = 5, which indicates that the number of steps forward was 5). Prognosis-related metabolic pathways with a relative importance >0.5 were selected. Regarding stemness, six stemness index (mDNAsi, EREG-mDNAsi, DMPsi, ENHsi, mRNAsi, and EREG-mRNAsi) were determined based on epigenetic data using a one-class logistic regression (OCLR) machine learning algorithm ([16](#_ENREF_16)). One index (mRNAsi) reflects gene expression, and another (mDNAsi) reflects epigenetic features. Pearson correlations of the m6A regulators with immune cell infiltration, metabolic pathways, and stemness index were calculated and the correlation matrix was visualized using the “pheatmap” R package ([17](#_ENREF_17)).

**Weighted gene co-expression network analysis**

To identify immune/metabolism/stemness phenotype-related genes related to the m6A modification patterns, WGCNA was performed using the “WGCNA” R package ([18](#_ENREF_18)). Biweight midcorrelation was used to assess correlations in the WGCNA. A scale-free network to select the most suitable β parameter for converting the adjacency matrix into a scale-free topology was generated (β = 9, R^2^ = 0.90). A soft-​thresholding power of 9 was set for network construction and module detection.

In a module–trait analysis, the module eigengene was related, with p<0.05 as the cutoff, to the following 12 phenotypes: ImmuneScore, five metabolic pathways (selected in the random survival forest analysis), and six stemness index. The “limma” R package ([19](#_ENREF_19)) was used to identify m6A modification-related DEGs, based on |log2(fold change [FC])|>0.5 and adjusted p<0.05, between the two m6A modification patterns. These DEGs were then used in the WGCNA.

**Identification of DNA methylation subgroups**

The CpG sites near the 21 m6A regulator genes were identified using the UCSC Genome Browser. Univariate Cox regression was used to identify methylation sites that were significantly (p<0.05) associated with STS patients’ survival and these sites were subjected to consensus clustering.

**Construction of m6Ascore**

We developed an m6A scoring scheme to quantify the m6A modification level of individual patients using a PCA algorithm. First, the DEGs between different m6A modification patterns were selected and the prognostic value of each gene was assessed using univariate Cox regression (p<0.05). We subjected the resultant prognostic genes to PCA, and principal components 1 and 2 were extracted. Thus, this method mainly focuses the m6Ascore on the largest blocks of well correlated (or anti-correlated) genes. We then used a formula to define the m6Ascore: m6Ascore = ∑(PC1*i* - PC2*i*), where *i* is the expression of prognostic m6A phenotype-related genes. Based on the correlation between m6Ascore and survival, a cutoff was determined using the “surv-cutpoint” function of the “survminer” R package, and the samples were divided into high- and low-m6Ascore groups. STS patients with m6Ascore greater than this cutoff were classified into the high-m6Ascore group, while STS patients with m6Ascore less than this cutoff were classified into the low-m6Ascore group.

**Tumor immunogenicity analysis**

The correlations of m6Ascore with the following immunogenicity biomarkers were assessed: TMB, neoantigen burden, DNA damage including homologous recombination deficiency (HRD), loss of heterozygosity (LOH; number of segments with LOH events, and fraction of bases with LOH events), intratumor heterogeneity (ITH), and aneuploidy were obtained from a previous study ([16](#_ENREF_16)). Additionally, the differences between the high- and low- m6Ascore groups were calculated.

**Immunotherapy response prediction**

We predicted the immunotherapy response for each STS sample based on the expression of 15 immune checkpoint-related genes. Additionally, a systematic search identified two immunotherapy cohorts that were used to further validate the value of m6Ascore for predicting immunotherapy responses. The first cohort was the IMvigor210 cohort ([10](#_ENREF_10)), which comprises muscle-invasive bladder cancer tissues from patients (n=348) treated with the anti-PD-L1 antibody atezolizumab (complete expression data and detailed clinical data were obtained from http://research-pub.Gene.com/imvigor210corebiologies). The second cohort was a longitudinal cohort ([20](#_ENREF_20)) of tissue samples from metastatic melanoma patients (n=47) treated with sequential immune checkpoint blockade (CTLA-4 blockade followed by PD-1 blockade at progression). In addition, clinical and transcriptomic data sets from patients with melanoma treated with ipilimumab immunotherapy in the TCGA-SKCM cohort were analyzed to determine the predictive value of the m6Ascore. The m6Ascore of each patient in the cohorts was calculated, and the differences in responses to immunotherapy/survival between the high- and low-m6Ascore subgroups in each cohort were compared.

Next, we used the Tumor Immune Dysfunction and Exclusion (TIDE) algorithm ([21](#_ENREF_21)) to predict responses to immune checkpoint blockade based on pre-treatment tumor profiles (by modeling tumor immune evasion via integrating the expression signatures of T cell dysfunction and T cell exclusion). The lower the TIDE score, the better the response to immunotherapy. We compared the difference of TIDE scores between the high- and low-m6Ascore groups and the correlation between m6Ascore and TIDE score.

We also used SubMap algorithm ([22](#_ENREF_22)) to compare the similarities of the expression profiles of the high- and low- m6Ascore groups in TCGA-SARC and GSE21050 cohorts with an independent dataset of 47 melanoma patients treated with immunotherapy. The lower the p values, the higher the similarity. Recommended default parameters, 1000 random permutations for Fisher’s statistics, were used. The “complexHeatmap” R package ([23](#_ENREF_23)) was used to visualize the SubMap results.

**Chemotherapy response prediction**

We predicted the chemotherapy response for each STS sample by training a predictive model on cell line data from the largest publicly available pharmacogenomics database (Genomics of Drug Sensitivity in Cancer [GDSC], <https://www.cancerrxgene.org/>) using ridge regression. As commonly used chemotherapeutic drugs for STS patients, docetaxel, doxorubicin, and gemcitabine were selected for analysis. A lower half-maximal inhibitory concentration (IC50), estimated by ridge regression, indicates a better sensitivity to a given drug. The prediction process was implemented using the “pRRophetic” R package. All parameters were set to default, the batch effect was removed using “ComBat”, tissue type was set to “allSoldTumours”, and duplicate gene expression was summarized as mean value.

**Verification of the utility of m6Ascore in pan-cancer analysis**

Data on pan-cancer cohorts were also downloaded from Genomic Data Commons (GDC) Pan-Cancer in the UCSC Public Hub for further analysis, including gene expression data (log2(TPM+1)), overall survival time, and survival status. The m6Ascore of 10327 samples of 32 cancer types was calculated by m6A scoring formula we defined. The correlations of m6Ascore with TMB, ImmuneScore, and MSI were analyzed in Spearman correlation analyses and visualized using the “fmsb” R package.

**Statistical analysis**

Correlations of the TME-infiltrating immune cells and expression of m6A regulators were computed using Spearman and distance correlation analyses. One-way analysis of variance (ANOVA) and Kruskal–Wallis tests were used to compare three or more groups. Survival analysis was performed using the “survival” R package. All statistical p values were two sided, with p<0.05 indicating statistical significance. An alluvial diagram was used to visualize the changes in the attributes of individual patients in different clusters. All data processing was conducted in R 4.0.1 software.

1. Mayakonda A, Lin DC, Assenov Y, Plass C, Koeffler HP. Maftools: efficient and comprehensive analysis of somatic variants in cancer. *Genome Res* (2018) 28(11):1747-56. doi: 10.1101/gr.239244.118

2. Zhang H, Meltzer P, Davis S. RCircos: an R package for Circos 2D track plots. *BMC Bioinformatics* (2013) 14:244. doi: 10.1186/1471-2105-14-244

3. Mermel CH, Schumacher SE, Hill B, Meyerson ML, Beroukhim R, Getz G. GISTIC2.0 facilitates sensitive and confident localization of the targets of focal somatic copy-number alteration in human cancers. *Genome Biol* (2011) 12(4):R41. doi: 10.1186/gb-2011-12-4-r41

4. Wang T, Kong S, Tao M, Ju S. The potential role of RNA N6-methyladenosine in Cancer progression. *Mol Cancer* (2020) 19(1):88. doi: 10.1186/s12943-020-01204-7

5. Wilkerson MD, Hayes DN. ConsensusClusterPlus: a class discovery tool with confidence assessments and item tracking. *Bioinformatics* (2010) 26(12):1572-3. doi: 10.1093/bioinformatics/btq170

6. Sturm G, Finotello F, Petitprez F, Zhang JD, Baumbach J, Fridman WH, et al. Comprehensive evaluation of transcriptome-based cell-type quantification methods for immuno-oncology. *Bioinformatics* (2019) 35(14):i436-i45. doi: 10.1093/bioinformatics/btz363

7. Yoshihara K, Shahmoradgoli M, Martínez E, Vegesna R, Kim H, Torres-Garcia W, et al. Inferring tumour purity and stromal and immune cell admixture from expression data. *Nat Commun* (2013) 4:2612. doi: 10.1038/ncomms3612

8. Hänzelmann S, Castelo R, Guinney J. GSVA: gene set variation analysis for microarray and RNA-seq data. *BMC Bioinformatics* (2013) 14:7. doi: 10.1186/1471-2105-14-7

9. Rosario SR, Long MD, Affronti HC, Rowsam AM, Eng KH, Smiraglia DJ. Pan-cancer analysis of transcriptional metabolic dysregulation using The Cancer Genome Atlas. *Nat Commun* (2018) 9(1):5330. doi: 10.1038/s41467-018-07232-8

10. Mariathasan S, Turley SJ, Nickles D, Castiglioni A, Yuen K, Wang Y, et al. TGFβ attenuates tumour response to PD-L1 blockade by contributing to exclusion of T cells. *Nature* (2018) 554(7693):544-8. doi: 10.1038/nature25501

11. Yu G, Wang LG, Han Y, He QY. clusterProfiler: an R package for comparing biological themes among gene clusters. *OMICS* (2012) 16(5):284-7. doi: 10.1089/omi.2011.0118

12. Mootha VK, Lindgren CM, Eriksson KF, Subramanian A, Sihag S, Lehar J, et al. PGC-1alpha-responsive genes involved in oxidative phosphorylation are coordinately downregulated in human diabetes. *Nat Genet* (2003) 34(3):267-73. doi: 10.1038/ng1180

13. Aran D, Hu Z, Butte AJ. xCell: digitally portraying the tissue cellular heterogeneity landscape. *Genome Biol* (2017) 18(1):220. doi: 10.1186/s13059-017-1349-1

14. Therneau T. A Package for Survival Analysis in R. R package version 3.2-3. *R package version 32-11,*[*https://CRANR-projectorg/package=survival*](https://CRANR-projectorg/package=survival) (2020).

15. Ishwaran H, Kogalur U, Kogalur M. RandomForestSRC: Fast Unified Random Forests for Survival. *Regression, and Classification (RF-SRC)[(accessed on 16 July 2020)]* (2021).

16. Thorsson V, Gibbs DL, Brown SD, Wolf D, Bortone DS, Ou Yang TH, et al. The Immune Landscape of Cancer. *Immunity* (2018) 48(4):812-30.e14. doi: 10.1016/j.immuni.2018.03.023

17. Kolde R, Kolde MR. Package ‘pheatmap’. *R package* (2015) 1(7):790.

18. Langfelder P, Horvath S. WGCNA: an R package for weighted correlation network analysis. *BMC Bioinformatics* (2008) 9:559. doi: 10.1186/1471-2105-9-559

19. Ritchie ME, Phipson B, Wu D, Hu Y, Law CW, Shi W, et al. limma powers differential expression analyses for RNA-sequencing and microarray studies. *Nucleic Acids Res* (2015) 43(7):e47. doi: 10.1093/nar/gkv007

20. Roh W, Chen PL, Reuben A, Spencer CN, Prieto PA, Miller JP, et al. Integrated molecular analysis of tumor biopsies on sequential CTLA-4 and PD-1 blockade reveals markers of response and resistance. *Sci Transl Med* (2017) 9(379). doi: 10.1126/scitranslmed.aah3560

21. Jiang P, Gu S, Pan D, Fu J, Sahu A, Hu X, et al. Signatures of T cell dysfunction and exclusion predict cancer immunotherapy response. *Nat Med* (2018) 24(10):1550-8. doi: 10.1038/s41591-018-0136-1

22. Hoshida Y, Brunet JP, Tamayo P, Golub TR, Mesirov JP. Subclass mapping: identifying common subtypes in independent disease data sets. *PLoS ONE* (2007) 2(11):e1195. doi: 10.1371/journal.pone.0001195

23. Gu Z, Eils R, Schlesner M. Complex heatmaps reveal patterns and correlations in multidimensional genomic data. *Bioinformatics* (2016) 32(18):2847-9. doi: 10.1093/bioinformatics/btw313
